# Millennium Development Goal Four and Child Health Inequities in Indonesia: A Systematic Review of the Literature

**DOI:** 10.1371/journal.pone.0123629

**Published:** 2015-05-05

**Authors:** Julia Schröders, Stig Wall, Hari Kusnanto, Nawi Ng

**Affiliations:** 1 Umeå Centre for Global Health Research, Division of Epidemiology and Global Health, Department of Public Health and Clinical Medicine, Umeå University, Umeå, Sweden; 2 Department of Public Health, Faculty of Medicine, Gadjah Mada University, Yogyakarta, Indonesia; National Cardiovascular Center Hospital, JAPAN

## Abstract

**Introduction:**

Millennium Development Goal (MDG) 4 calls for reducing mortality of children under-five years by two-thirds by 2015. Indonesia is on track to officially meet the MDG 4 targets by 2015 but progress has been far from universal. It has been argued that national level statistics, on which MDG 4 relies, obscure persistent health inequities within the country. Particularly inequities in child health are a major global public health challenge both for achieving MDG 4 in 2015 and beyond. This review aims to map out the situation of MDG 4 with respect to disadvantaged populations in Indonesia applying the Social Determinants of Health (SDH) framework. The specific objectives are to answer: Who are the disadvantaged populations? Where do they live? And why and how is the inequitable distribution of health explained in terms of the SDH framework?

**Methods and Findings:**

We retrieved studies through a systematic review of peer-reviewed and gray literature published in 1995-2014. The PRISMA-Equity 2012 statement was adapted to guide the methods of this review. The dependent variables were MDG 4-related indicators; the independent variable “disadvantaged populations” was defined by different categories of social differentiation using PROGRESS. Included texts were analyzed following the guidelines for deductive content analysis operationalized on the basis of the SDH framework. We identified 83 studies establishing evidence on more than 40 different determinants hindering an equitable distribution of child health in Indonesia. The most prominent determinants arise from the shortcomings within the rural health care system, the repercussions of food poverty coupled with low health literacy among parents, the impact of low household decision-making power of mothers, and the consequences of high persistent use of traditional birth attendants among ethnic minorities.

**Conclusion:**

This review calls for enhanced understanding of the determinants and pathways that create, detain, and overcome inequities in child health in resource constraint settings like Indonesia and the promotion of actionable health policy recommendations and tailored investments.

## INTRODUCTION

In 2000, the 193 United Nations member states and 23 international organizations signed the Millennium Declaration and thus committed themselves to achieve eight Millennium Development Goals (MDGs) by the year 2015. The MDGs represent the biggest commitment in history to roll back global poverty and disease. Three of these goals (MDG 4, 5, and 6) are directly related to health. The fourth goal (MDG 4) is targeted at reducing by two-thirds, between 1990 and 2015, the mortality in children younger than five years [1]. Infant and child mortality rates are key indicators of child health and the overall socioeconomic development of a country. Even tough child mortality dropped worldwide by 41% (from 87 to 51 deaths per 1,000 live births between the years 1990 to 2011), it is still unacceptably high in many parts of the world. In 2011, 6,9 million children under the age of five died from mostly preventable causes. Only 27 out of 138 countries are projected to eventually achieve MDG 4 in 2015 [2, 3]. Four out of every five deaths below the age of five continue to occur in Sub-Saharan Africa and Central and Southern Asia. With the current speed of annual reduction in under-five mortality (3.9%) it will take until the year 2028 until MDG 4 is achieved globally [4]. Despite strong global efforts and accumulated evidence of what causes, prevents, and treats common childhood diseases, it remains a challenge to apply these lessons learnt and reduce child mortality in many low- and middle-income country (LMIC) settings.

It has been argued that achieving MDG 4 does not necessarily imply that the overall tenor of the Millennium Declaration (namely to address the development needs of the most underserved and disadvantaged segments of society) is met. The MDG 4 indicators are “equity blind” as they are simply the raw averages of a country’s national under-five mortality or immunization rates and unable to distinguish between a “just” and “unjust” distribution of the burden of child mortality among different population groups within each country [5]. Particularly with the soon approaching deadline of December 31^st^ 2015 there is a growing concern that persisting health inequities and unfavorable social determinants of health (SDH) might be major factors that eventually prevent the achievement of the MDG 4 targets in many countries. For instance, a 2010 UNICEF report revealed that in developing countries showing overall reductions in under-five mortality, inequities in child mortality outcomes between the poorest and the richest households still persisted by more than ten per cent [6].

National statistics show that child health is steadily improving in Indonesia and the country is currently regarded to be on track to achieve MDG 4 as it is increasing the measles immunization rate among one-year olds and reducing its under-five mortality rate from 97 to 32 as well as its infant morality rate from 68 to 23 deaths per 1,000 life births until 2015 [7]. But progress is not universal and on sub-national level Indonesia shows wide intra-country disparities in many child health indicators. This might indicate inequitable access to and provisions of health services across its provinces and districts and is not always translated into the aggregated national-level statistics on which MDG 4 relies [8]. Unavailability of reliable data sources remains as an obstacle for measuring the progress of MDG 4, not only due to Indonesia’s vast geographical and socio-demographic characteristics and recurrent natural and political crises but foremost due to the lack of health information systems and the lacking awareness to establish and maintain such systems. Achieving MDG 4 in an equitable manner will be a major challenge for both researchers and policy makers until 2015 and beyond. In light of this, the aim of this systematic review is to map the situation of MDG 4 with respect to disadvantaged populations in Indonesia applying the SDH framework. The specific objectives are to answer the following: *Who* are the disadvantaged populations? *Where* do they live? And, *why* and *how* is the inequitable distribution of children’s’ health explained in terms of the SDH framework?

## METHODS

A systematic review based on the PRISMA-Equity 2012 statement (Preferred Reporting Items for Systematic reviews and Meta-Analysis with a focus on health Equity) was adapted to guide the methods of this study [9] (S1 PRISMA Checklist). To our knowledge, a similar systematic review on child health inequities in Indonesia does not exist. This review is part of the EPI-4 project (Evidence for Policy and Implementation) designed to increase awareness and improve scientific evidence for an equitable achievement of the health-related MDGs 4, 5 and 6 in China, India, Indonesia and Vietnam [10].

### Definitions

**Fig 1:**
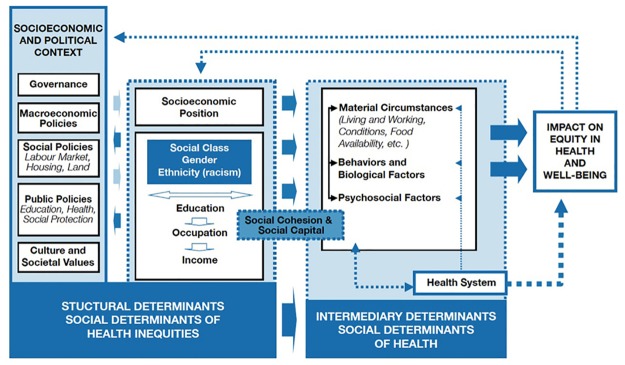
WHO Social Determinants of Health Framework.

The dependent variables of interest were MDG 4-related indicators such as under-five mortality, infant mortality, neonatal mortality and the immunization status of under-five year-olds. The majority of child deaths occur among so-called “disadvantaged populations”. Those are per definition denied benefits or services to achieve good health because of “irrelevant” characteristics [11] or they simply lack the capability to achieve health due to inadequate social arrangements [12]. Inequities in health are the consequence of an unfair distribution of power, money, resources and finally health services as well as the overall conditions of everyday life which constitute the SDH—in other words: the conditions in which people are born, grow, live, work, and age [13, 14]. This independent variable “disadvantaged populations” was defined by different categories of social differentiation using the mnemonic “PROGRESS” which is an acronym for Place of residence, Race/ethnicity, Occupation, Gender, Religion, Education, Socioeconomic status and Social capital [15]. These equity-related “PROGRESS” categories were particularly chosen based on their relevancy to the SDH framework (Fig 1) launched by the WHO Commission on Social Determinants of Health in 2005 [13].

### Sources

This review considered both electronic databases and the gray literatures. Three databases were searched: PubMed (http://www.ncbi.nlm.nih.gov/pmc/), Popline (http://www.popline.org) and JSTOR (http://www.jstor.org) to cover biomedical, reproductive health, and the social sciences / humanities literatures, respectively. Gray literature was manually searched for from databases of Indonesian governmental institutions and international non-governmental organizations. A detailed search syntax with key words used is presented in the supporting information (S1 Search Syntax). We included only publications in English and those published during 1995-May 2014.

### Eligibility Criteria and Identification of Studies

Eligibility for inclusion was restricted to the following criteria and fulfilling only one exclusion criterion was sufficient to exclude a study from the review:

- Study Site: Indonesia (national, provincial and district level studies, including multi-country studies where Indonesia was included).
- Design: Quantitative observational studies with a case-control, cross-sectional or cohort design, descriptive studies as well as qualitative studies were included; reviews and systematic reviews as well as economic evaluation and any experimental studies such as RCTs or clinical trials were excluded. Conference papers and proceedings, working papers, project descriptions and editorials or commentaries were also not eligible for inclusion.
- Outcome: an article should address any MDG 4-related indicator as described above.
- Relatedness: an article should address any of the components of the SDH framework (structural determinants or intermediary determinants); or it should include any cause, determinant, risk factor, or other explanation for child health inequity; or it should address one or more disadvantaged group(s) or one or more factor(s) that cause a disadvantage.

In the first level of screening, titles, abstracts and keywords were screened according to the eligibility criteria mentioned above. In the second level, full text articles of studies fulfilling the first screening level criteria were obtained and evaluated to be included in the review. Two independent reviewers (J. S. and N. N.) reviewed all titles, abstracts and full-texts; disagreements were settled by consensus to the other co-authors (S.W. and H. K). The reference management software EndNote version X6 was used to import and manage all literatures for this review.

### Data Analysis

In this study, the selected studies were analyzed following the guidelines for deductive content analysis. Deductive content analysis is used when the structure of analysis is operationalized on the basis of existing knowledge, theories, or models [16]. In our study the SDH framework served as the point of departure. The type of determinant represents the basis for the categorization and coding process. We used content analysis because it is a widely used qualitative research technique in public health researches and it provides an opportunity to make replicable and valid inferences from data to its context and to construct a practical guide to action for policy makers [17]. Because a meta-analysis was not possible to perform (due to the heterogeneity of the results) we chose to present the findings as a narrative synthesis [18].

## RESULTS

### Flow of included studies

**Fig 2:**
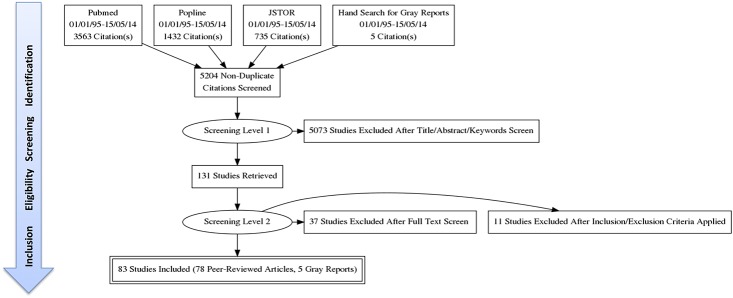
Flow of Included Studies.

Our search returned a total of 5204 studies from three databases. After applying the first level of screening, 131 studies were identified as potentially relevant. When applying the second screening level, 83 studies (78 peer-reviewed articles and 5 gray reports) were identified and included for data extraction and analysis. Of the 78 included studies, ten applied a qualitative study design while 68 used quantitative methods. The whole flow of included studies according to the PRISMA-Equity guidelines is shown in Fig 2. In this section, we will present the results for each structural (place of residence, income, education, gender, ethnicity) and intermediary (material circumstances, behaviors and biological factors, psychosocial, health care system) determinant of child health based on the SDH framework (Fig 1). In this sense, we do not treat structural and intermediary determinants as independent constructs but rather as dependent ones by nesting the results on intermediary determinants within the structural ones.

### Place of residence

Our review identified 40 studies dealing with how the type of community, i.e. rural or urban, influences child health via a set of intermediary determinants such as material circumstances, behaviors and biological factors, psychosocial factors and the health care system. This is summarized in “Table 1”. Our results show that even though the rural-urban gap in child health inequalities decreased over time, the under-five survival in the peripheral remote areas in the outer islands still lag behind the progress made by urban areas in the central Java-Bali regions. The malfunctions within the rural health care system stand out as one of the most prominent social determinants of child health inequities in Indonesia.

**Table 1:** Place of Residence and Child Health in Indonesia.

	Material Circumstances	Behaviors and Biological Factors	Psychosocial Factors	Health System
***Rural***	Poor household and community infrastructure [19–22]; Malnutrition [22–26]	Low vaccination uptake [7, 29–35, 41]; Low nutritional supplementation [36, 37, 40]; High exclusive breastfeeding [38–40]	Non-acceptance of childhood immunization [35]	Long distance to health facilities [7, 19, 30–32, 41–49]; Low quality of health facilities [35, 48, 50–54]; High number of inactive or closed clinics [21, 42, 43]; Low utilization of services [7, 30–33, 35, 36, 41–45, 51, 55–57]
***Urban***	Double burden of malnutrition [27, 28]	Low exclusive breastfeeding and malnutrition [39]	*No studies found*	*No studies found*

#### Material circumstances

Environmental determinants have been identified as a leading contributor to rural child health. Four records found an association between poor household and community infrastructures such as the lack of toilet facilities and piped sewage systems, absence of waste disposal systems or non-access to clean piped water and poor child health in rural Indonesia [19–22].

Mellington and Cameron showed that having a toilet and having piped water significantly decreases the probability of child mortality in rural settings by 6.2% and 3.3%, respectively [19]. A similar conclusion was drawn from a study by Semba et al., which characterized the relationship between the presence of an improved latrine with diarrheal disease and under-five mortality. The study showed that in rural families open defecation and unimproved latrines are closely associated with a history of diarrhea and under-five child mortality; i.e. more than half of the households in rural Indonesia do not have an improved latrine and every tenths of these have experienced a history of under-five mortality [20]. A study by Paknawin-Mock et al. showed the contribution of community-level services and infrastructure on early childhood growth among tea plantation communities in Bandung in West Java and concluded that environmental sanitation—foremost the quality of community water sources and garbage standards had a positive effect on child growth and development [21]. A report by Statistics Indonesia (Badan Pusat Statistik, BPS) likewise pointed out that despite the fact that universal access to safe drinking water and environmental sanitation has been defined as national developing goals, large differences between rural and urban households still persist conceding rural households at a greater disadvantage [22].

Our review also identified four sources, which argued that living in rural settings poses a risk factor for malnutrition [23–26]. The studies by Sari et al. [23] and Semba et al. [24]—both based on data from the 2003 wave of the Nutritional Surveillance System (NSS) found that the prevalence of stunting—chronic malnutrition manifested in growth failure, is higher in rural areas (range 30%–50%)—than in urban slum areas. This might indicate that the quality and quantity of foods provided for children varies substantially across the Indonesian archipelago. Another study by Ng et al. explored complementary feeding indicators and determinants of poor feeding practices based on the 2007 Indonesian Demographic and Health Survey (IDHS) and found that there are vast geographic differences regarding children’s dietary diversity. For instance, infants living in Java and Bali were less likely to be fed more than four different food groups compared to infants from outer Java and Bali indicating that there are different food cultures or beliefs about which foods are appropriate for infants in different rural regions in Indonesia [25]. According to two studies, there was a higher prevalence of moderate and severe under nutrition, inadequate iodized salt consumption, and lower consumption of micronutrient-rich foods in rural Indonesian settings [22, 26]. The later was especially difficult to access and afford in remote rural settings. In contrary, Doak et al. and Oddo et al. identified a strong urban effect on the existence of a dual burden of malnutrition—the coexistence of over- and underweight individuals in the same household, in Indonesia [27, 28]. Urban households were three times more likely to be affected by dual burden of malnutrition compared to their rural counterparts [27]. The double burden of malnutrition phenomenon is, however, spreading across rural areas as they progress economically, putting especially stunted children with overweight mothers at particular risk [28].

#### Behaviors and biological factors

A body of research has been published on child health promoting behaviors such as vaccination and immunization, uptake of nutritional supplementation, and compliance to recommendations for breastfeeding among rural Indonesians [22, 26, 29–40].

Indonesia’s 2004, 2010 and 2011 MDG achievement reports presented a persisting rural-urban gap in childhood vaccination uptake [29, 30]. The percentage of 1-year-old children immunized against measles increased from 45 to 87 percent from 1991 to 2011. Yet in 2011, 18 provinces felt below the national measles vaccination coverage rate of 75%. Rural settings in North Sumatra, Aceh and Papua showed only a coverage rate ranging from 30 to 50% whereas almost 100 per cent of children received a timely measles vaccination in the Yogyakarta Special Region [7]. Other studies identified that in 16 out of 33 provinces, the first dose of measles vaccination was administered to only 69% of children in rural areas in contrast to more than 80% of children in urban settings. The greatest differentials have been ascribed to Maluku and Papua which are predominantly rural regions with high poverty rates [32]. Further, Fernandez et al. showed that the vaccination coverage for all twelve childhood vaccinations listed in the Indonesian vaccination plan was significantly higher among urban children [31, 32]. Despite varying numbers on the provision of immunization to children in different data sources, it remains evident that there is great need to introduce measures that will close the persistent rural-urban gaps for childhood vaccination uptake especially for tetanus vaccinations [33], hemophilus influenza type b and pneumococcal vaccinations to prevent deaths due to acute respiratory infections (ARI) and central nervous system infections among infants [34].

While the majority of studies dealt with vaccination coverage, a study by Serquina-Ramiro et al. investigated in which way the type of community influences parents’ acceptance of childhood immunizations. The authors revealed that parents from urban areas or parents migrating from rural to urban settings were more likely to accept vaccinations and use services and programs more often than parents who had only access to the limited resources of the restricted social networks in rural villages [35].

Two articles researched on the impact of iron and folic acid (IFA) and Vitamin A nutritional supplementation during pregnancy on child mortality in rural and urban settings in Indonesia [36, 37]. Dibley et al. [36] and Titaley et al. [37] identified a regional variation in the risk of death during the first five years with higher mortality in rural compared to urban areas. The mortality differentials could be attributed to the protective effect of IFA taken by urban mothers during pregnancy that lowers the risk of neonatal, infant and under-five deaths by 39, 34 and 34%, respectively.

The advantages of exclusive breast-feeding (EBF) and the risks of morbidity and mortality from untimely use of breast milk substitutes among children have been well documented for Indonesia. About 40% of all infants younger than 6 months have been exclusively breastfed in Indonesia. EBF was less frequent in urban areas compared to rural areas. About every fifth infant received formula milk and 45% received complementary foods in rural areas in Indonesia, compared to 52% and 67% respectively in urban areas [39]. Yet, two out of five breastfed infants in Indonesia suffered from micronutrient deficiencies and anemia [38, 39]. A study by Dijkhuizen et al. pointed out that breast milk poses a key-connecting factor between the micronutrient status of lactating mothers and that of their breastfed infants. This is of special importance when the mother already suffers from conditions such as Vitamin A, iron and zinc deficiencies and thus posed a risk for transferring nutrient deficiencies or anemia onto the child [40]. This phenomenon of coexisting micronutrient deficiencies among mothers and children was both common in rural and urban Indonesia but has different causes: in rural areas this is caused by high EBF rates and low nutritional supplementation among mothers and children while in urban areas the early termination of EBF and the high use of commercial foods constitutes missed opportunities for health benefits and mortality reductions deriving from EBF [38]. In sum, suboptimum new-born and infant feeding practices and the subsequent effects on child health differed in rural and urban populations and requires different attention from health care workers, food industry and policy makers [39].

#### Psychosocial Factors

We could only identify one study on the influence of psychosocial factors such as peer pressure and the presence of high social cohesion on measles immunization acceptance among mothers [35]. The study revealed that the majority of mothers in rural villages in Purworejo district in Central Java, who got their child vaccinated (87%) and those with unimmunized children (70%), felt pressured or forced in their decision-making process. Village heads, religious leaders, neighbors or traditional midwifes play an important role in parent’s decision-making process whether to (or not to) accept immunization for their children. Close social relations, less access to resources and restricting traditional norms predominantly characterized rural areas. Urban areas include wider social networks and information resources, which can positively affect childhood immunization rates.

#### Health Care System

The relationship between child health inequities and characteristics of the Indonesian rural health care system, such as access and availability of health centers, the quality of services, and the utilization of care, is documented by nearly 30 publications.

The Indonesian government gave continuous high priority to increase the number of community health care centers in remote areas in order to tackle the persistent low availability and accessibility of services for rural populations [7, 29, 30, 41]. Several studies identified that a long physical distance coupled with poor infrastructure and time constraints were strong predictors for children missing out Vitamin A supplementation, diphtheria-pertussis-tetanus (DPT) immunizations, oral poliovirus (OPV) immunizations [42, 43], measles vaccinations [31, 32, 42], or not receiving postnatal care services [44–49]. One study stated that adding 100 extra health care centers in rural provinces would decrease the probability of a mother losing a child by only 1.1 percentage points whereas urban provinces would benefit almost ten times more [19]. This illustrates how persistent child health inequalities between rural and urban settings in Indonesia are, and how little benefit comes from a greater quantity of health facilities if people remain geographically isolated from them.

Lack in quantity and availability, of trained health staff as well as structural and internal quality of facilities are major obstacles for child health in rural Indonesian settings. A physician in Indonesia is on average responsible for 6000 patients, and the ratio is worse in rural area. This constitutes the second lowest physician-to-population ration in the Asia Pacific region [50]. Major obstacles for this staffing gap are insufficient incentive packages to attract physicians, nurses and midwife to work in remote areas [50]. When the Indonesian government enacted a zero-growth policy in 1992, the number of facilities lacking a medical doctor and a trained midwife increased in the rural outer Java-Bali regions [51]. However, the number of nurses did not decrease but many nurses only received insufficient training and material resources to offer good quality care [51, 52]. Moreover, there has been an imbalance in the number of public and private health care providers in rural compared to urban areas. Children from rural regions were more likely delivered or treated by non-formal health care providers such as a traditional birth attendant (TBA), or at public facilities compared to children from urban areas that receive, presumably, higher quality care from the private sector [48, 53, 54]. Many rural health posts—the *posyandus*, were closed or not active [21, 42, 43]. This has been identified as a leading cause for children missing out on crucial survival interventions [42].

Three studies have researched the structural and internal quality of rural health care services and facilities in Indonesia [35, 51, 52]. Many public facilities lacked clean water sources, functioning instruments such as microscopes or means to sterilize equipment [51]; misuse and overuse of pharmaceuticals and utensils has also been reported [52]. Lost opportunities for childhood vaccinations have been manifold in rural settings due to problems regarding stocking, transportation and organization of immunization services. Only one-third of health centers have essential vaccines in stock [51]; in many cases the cold chain lasts until the community health center—the *puskesmas*, or the sub-health center—the *pustu*, but rarely until the midwife, who is the first contact point to the village population [35, 51].

Unavailability, poor access and poor quality of rural health care services subsequently led to low utilization of child health care in rural Indonesia [7, 30, 33, 41]. Common examples for low service utilization in rural settings were low usage of ante- and postnatal care [7, 30, 41, 44–46], low uptake of vaccination and nutritional supplementation programs [31, 32, 35, 36, 42, 43, 51, 55]. It has also been reported that in many rural villages, there were ten times more traditional birth attendants compared to midwives. Consequently, outpatient care and institutional deliveries with skilled birth attendants are outnumbered by home deliveries assisted by unskilled traditional birth attendants putting children and likewise mothers at higher risk of morbidity and mortality [48, 49, 56]. One study showed that the introduction of a maternal and child health handbook (MCHH) was an effective measure to increase service utilization for pre- and postnatal care and to establish a continuum of care for mothers and newborn children in rural areas in Indonesia [57].

### Income

Our review has identified 51 studies that provide information on how higher income and higher social status are linked to better health or—vice versa, how material deprivation and poverty construct poor health through different intermediary determinants. These results are shown in “Table 2”.

**Table 2:** Income and Child Health in Indonesia.

	Material Circumstances	Behaviors and Biological Factors	Psychosocial Factors	Health System
***Low Income***	Malnutrition and food insecurity [23, 26, 60–70]; Poor household infrastructure and lack of facilities [19, 71]; Reduced child care [59, 70, 72]	Nutritional status and parental smoking [20, 23, 65, 69, 77–80]; Reduced child care [25, 36, 44, 45, 61, 75, 76]	High social cohesion and psychosocial stress [71, 73, 74]	Less utilization and low quality neonatal and child curative health care [7, 29, 30, 33, 35, 36, 41, 45–49, 51, 53, 81, 82]; Low consultation of skilled birth attendant and home births [32, 33, 47–49, 84]; Discriminatory behavior of health care staff [51, 85–87]
***High Income***	Overweight and double burden of malnutrition [28, 73, 74]	Less breastfeeding and increased bottle-feeding [25, 38, 73–75]	*No studies found*	*No studies found*

Our review supported the general notion that the greater the gaps between the richest and poorest people are, the greater the differences in health are. Many studies could observe a socioeconomic gradient in health even after adjusting for proxies of socioeconomic status such as education. We are not aware of any studies dealing with a reverse causation, i.e. a loss of income because parents have to stay at home to take care of ill children. Also, we could only find very little evidence how occupation—i.e. risky types of labor, occupational hazards, working in the informal sector, or being unemployed, influenced child health in Indonesia [58, 59]. Food poverty and food insecurity related to material deprivation stemmed out as one of the most prominent social determinants of child malnutrition and ill health in Indonesia.

#### Material circumstances

Many studies showed that child malnutrition and food poverty and insecurity were directly linked to household material circumstances as well as the food system at macro level. Food insecurity arises when there is no physical or socioeconomic access to sufficient, safe and nutritious foods that meet the dietary needs and individual food preferences. Food poverty can emerge due to either the unavailability of food, inadequate and low purchasing power, or inappropriate utilization at the household level. In Indonesia it was shown that low purchasing power leads to the consumption of fewer meals per day [60, 61] and to low-quality food consumption or low-quality supplementary foods consumption [26, 62–64]. Greater expenditure on fruits and vegetables and animal-source and non-grain foods lowered the risk of child stunting and is associated with less under-five mortality [23, 65]. Several studies by Semba et al. stated that non-utilization of iodized salt increases the risk of malnutrition and child mortality [66], that non-consumption of iron-fortified foods such as milk and noodles increase the risk of stunting and anemia [67, 68], and that the inability to purchase safe drinking water was associated with child morbidity and mortality, especially in urban slums [69]. Bardosono et al. stated that reducing the quantity and quality of food consumption are strong determinants of child malnutrition during economic crises where already deprived households are put on additional risks [70]. Poor household living conditions and infrastructures arising as a consequence of low socioeconomic status were also pervading structural determinants of child health in Indonesia. A lack of basic household facilities such as a toilet, piped drinking water or a waste disposal system was mentioned by two studies [19, 71]. However, in line with results presented earlier, high income and thus a higher weekly per capita expenditure on food could pose risks for the emergence of a dual burden of malnutrition [28].

We could also identify how difficulties in combining childcare and work affected child health. In Indonesia, women are considered the primary caregiver to the child. Still, around 60% of mothers in Central Java are involved in income generating work [72]. Maternal employment outside the house can be protective for child health since it raises the household income and the general welfare of the family. However, several studies have reported that having a working mother was also positively related to higher prevalence of stunting and anemia [70] or that children of mothers who had informal work were at higher risk for malnutrition [59]. Toyama et al. and Gryboski et al. claimed that in order to improve child health and welfare, policies should specifically target families with a working mother—the so called “poverty avoider” group, and non-maternal caregivers [59, 72]. Roshita et al. pointed out the need for further research on how to support Indonesian mothers across a range of socioeconomic and income strata with increased options for quality child-care arrangements and child feeding practices especially through the dissemination of information on both underweight and overweight [73, 74].

#### Behaviors and Biological Factors

Our review identified evidence on how parental economic conditions influence health behaviors—especially in the form of care taking and care seeking and ensuring an adequate child nutritional status. In Indonesia, low income is a strong predictor for poor feeding practices [25, 75], non-utilization of IFA supplementation during pregnancy [36], or reduced care seeking in the pre- and postnatal period [44, 45, 71]. We also obtained evidence that high-income also contributes to poor child nutritional statuses as mothers from higher income families terminate the breastfeeding period and initiate bottle-feeding much earlier compared to lower-income mothers [25, 73–76].

The majority of published literature on behavioral factors focused on paternal smoking and its consequences for child health. The prevalence of smoking especially among men remained high in South East Asia, and it is about 60% in Indonesia. Parental smoking influences child health, not only through environmental tobacco smoke exposure and increased risk of respiratory illnesses, but also through diverting household income from food to cigarettes. The amount of household money spent on cigarettes is associated with proportionally lower expenditures on basic necessities such as food, safe drinking water, other household facilities and children’s education. Paternal smoking also increased the risk of chronic malnutrition and food insecurity [23, 77–79] and consequently increased the risk of under-five mortality [65, 69, 80]. The population-attributable risk of excess under-five child mortality due to smoking ranged from 14% in urban slums to 24% in rural areas, which accounted for 90 child deaths per day in Indonesia [80].

#### Psychosocial factors

We could only find three studies that provided evidence on the impact of low income on parental psychosocial stress. Poerwanto et al. claimed that economic disadvantages increase the risk of infant mortality through a lack of social cohesion (which arises from the gap between the rich and the poor) and fostering the adoption of ill health behaviors among parents such as the absence of family planning, early childbearing age, and short birth intervals [71]. Studies by Roshita et al. showed that maternal self-confidence regarding the knowledge about child nutrition, cooking skills, or the knowledge about where to purchase foods are strong determinants of good child nutritional status [73, 74].

#### Health system

The relationship between child health inequities and healthcare quality, utilization, and affordability among the poor is well documented for Indonesia. In general, quality of care for children—especially hospital care—are in strong need of improvement cycles [81]. Low income and financial constraints were strongly associated with a low utilization of quality care for both mothers and children. Several studies showed a general low accessibility to inpatient and outpatient health care [29, 30, 33, 51, 82, 83] among the poor segments of society. Studies by Ramiro et al. and Fernandez et al. also reported on differential measles vaccination uptake in low-income groups and Dibley et al. reported less IFA supplementation uptake among the poor [32, 35, 36]. Financial constraints have been shown to be strong predictors for under-utilization of antenatal care services and non-utilization of postnatal care services [45–49]. Economic reasons were the most prominent factors affecting women’s decision to use professional delivery care services. Low consultation of skilled delivery services and a high prevalence of home births attended by traditional birth attendants were discussed in several studies [32, 33, 47–49, 84]. For example, a qualitative study conducted through focus group discussions in six villages in West Java Province revealed that financial limitations were the major constraint that prevented women from accessing hospital institutional delivery or trained birth attendants. It is noted that a hospital delivery costs around 2 million Indonesian Rupiah (IDR) (approximately 160 USD) whereas a traditional birth attendant costs only around IDR 50.000—which accounts for less than 5 USD [48].

Only little evidence was available on barriers due to discrimination and corruption towards the poor. One study suggested evidence for facility-based discrimination or corruption in form of informal user fees against the poor for pediatric and prenatal care [51]. Discriminatory attitudes, beliefs and behaviors of health care workers towards low-income family members have been reported by three studies [85–87].

### Education

Our review found that low educational attainment was strongly associated with low health literacy—i.e. low ability to gather, understand and use health and health care related information. We identified 39 records that showed the association between low parental educational attainment and child health. These results are displayed in “Table 3”. Our review supports the general notion that education highly influences health-promoting behaviors and fosters better-informed decision making in terms increased hygiene, public health or nutritional knowledge and the adoption of health-related recommendations such as vaccination or supplementation uptake. Thus, health illiteracy stemmed out as one of the most prominent social determinants of child mortality and morbidity in Indonesia.

**Table 3:** Education and Child Health in Indonesia.

	Material Circumstances	Behaviors and Biological Factors	Psychosocial Factors	Health System
***Low Education***	Malnutrition, food insecurity [23, 61, 65, 71]	Lack of hygiene and public health knowledge [19, 24, 63, 88, 89, 97]; Low nutritional knowledge and unhealthy food practices [60, 66, 69, 88, 90–92]; Smoking [78–80]; Low vaccination and supplementation uptake [31, 32, 35–37, 42, 43, 49, 93, 94]; Low pregnancy and childhood disease knowledge [44, 58, 89, 94, 95]	Social pressure [94]	Miscommunication and misconceptions about treatment and less use of modern health care services [22, 42, 51, 93, 94]; Discrimination by health care workers [51, 86]; Low health care decision making by women [98]
***High Education***	*No studies found*	Less breastfeeding [38, 75, 76]	*No studies found*	*No studies found*

#### Material circumstances

Several studies have shown that the probability of infant mortality attributable to low maternal education works through the pathways of material deprivation, e.g. households with less educated parents spent less money on healthy food groups which led to child malnutrition [23, 61] and mortality [65, 71]. For instance, a study by Poerwanto et al. showed that the risk of infant death was threefold higher among less educated mothers compared to mothers with more than seven years of education [71].

#### Behaviors and biological factors

In Indonesia, education can be a good proxy of health literacy. That can represent itself in various forms; i.e. knowledge and practice of personal hygiene [19, 24, 63, 88, 89] or nutritional knowledge [60, 88, 90–92]. Several studies investigated the association of education and the use of iodized salt [66], clean drinking water [69] or other protective childcare behaviors such as non-smoking (especially among fathers) [78–80]. Low health literacy in many instances resulted in lower vaccination uptake [31, 32, 35, 42, 43, 93] or in a lack of understanding of the benefits of Vitamin A supplementation [43] or IFA supplementation [36, 37, 49]. Several studies have also shown the lack of a clear understanding of the danger signs during pregnancy and immediately after birth [44, 94] or lack of knowledge on common childhood diseases such as diarrhea [58, 89] or anemia [95]. High health literacy among parents led to increased utilization of modern obstetric services and trained birth attendants [96] and also contraceptives, whose use has been shown to increase the likelihood of prolonged breastfeeding periods past the age of one [97]. A study by Yanuarti et al. showed that breastfeeding is strongly related to children’s’ language, visual and motor development [92] but it has also been shown that higher educated women perform less exclusive breastfeeding [38, 75, 76].

#### Psychosocial factors

Low health literacy can also lead to psychosocial stress among parents. One study demonstrated that it is very likely that social censorship and gossip from family members or community members will maintain low health literacy among parents. For instance, a mother who experienced a child death and was blamed by family or community members for the loss will very likely maintain her level of health literacy for the next pregnancy [94].

#### Health system

Low education, and thus low health literacy, results very often in a lack of understanding about the nature and importance of any clinical treatment the child should receive. Many parents with lower educational level were unable to obtain and understand information from health care staff [22, 42, 51, 93, 94]. Such miscommunication led to misconceptions about for example the use of vaccines as a curative—not preventive measure [35, 86, 94]. Less educated mothers only possessed a limited health care decision making power, used less antenatal and delivery care, and their children were less likely to benefit from other primary care or preventive care programs later on [42, 43, 50, 51, 94, 98].

### Gender

The association between gender empowerment and child health is rather unexplored in Indonesia. Only three studies considered gender as a structural determinant of child health and in most cases the gender role of the mother played a bigger role than the gender of the child itself. All results are shown in “Table 4”.

**Table 4:** Gender Empowerment and Child Health in Indonesia.

	Material Circumstances	Behaviors and Biological Factors	Psychosocial Factors	Health System
***Not Empowered***	*No studies found*	*No studies found*	*No studies found*	Low household decision making power and low community participation of mothers [98–100]
***Empowered***	*No studies found*	*No studies found*	*No studies found*	*No studies found*

#### Material circumstances

No studies found

#### Behaviors and biological factors

No studies found

#### Psychosocial factors

No studies found

#### Health system

We could only find little evidence on how cultural gender norms impact on child health in Indonesia. One study showed that low household decision-making power hinders women to access child health care services [98]. Another study on the association between maternal community participation and child health described how mothers who actively participate in the community will engage in processes of information sharing and thus gain reciprocal advice on how to maintain their children’s health [99]. Moreover, community participation was able to increase the access and use of the health care services [100]. This reasoning is based on a social capital framework, arguing that if mothers invest in social relations they expect that this investment will generate an advantage, i.e. improved or maintained child health.

### Ethnicity

Like gender, ethnicity as a structural determinant of child health is also under-researched in Indonesia. Our review could only identify four studies that touch upon ethnicity-related inequities in child health. These results are shown in “Table 5”.

**Table 5:** Ethnicity and Child Health in Indonesia.

	Material Circumstances	Behaviors and Biological Factors	Psychosocial Factors	Health System
***Minority Groups***	*No studies found*	Preference for traditional healer [34, 52]; Home treatment and self-medication [52]; Home deliveries and use of traditional birth attendants [54]; Nutritional care [101]	*No studies found*	Low accessibility and affordability of modern health care facilities [52]
***Majority Groups***	*No studies found*	*No studies found*	*No studies found*	*No studies found*

#### Material circumstances

No studies found

#### Behaviors and biological factors

Two studies found that parents belonging to an ethnic minority group like the *Sasak* ethnic group in East Lombok were more likely to refer to a traditional healer (“belian”) within their community or practice self-medication when their children turn sick [34, 52]. The main reason for this was the lack of biomedical understanding and the subsequent greater social comfort within the traditional sector compared to the modern health care system. However, it has been observed that the “belian” is mostly visited for infant care while older children are frequently brought to a clinic when turning sick [52]. Another study showed that older mothers and those who belong to a non-Muslim minority are more likely to refer to a traditional birth attendant and give birth at home, which has implications for the survival of both mother and child [54]. One study found an association between care practices among *Karu* and *Minangkabau* ethnic minorities and child malnutrition—foremost undernutrition and wasting and stunting [101]. The study operated on the notion that food intake depends not only on food availability but also on the social interaction between parent and child.

#### Psychosocial factors

No studies found

#### Health system

We could only find one study providing information on the association between accessibility and affordability of the modern health care services and child mortality among ethnic minority groups. Grace et al. noted two important determinants for non-utilization of modern health care by ethnic minorities: first, the traditional healer is temporally and spatially more accessible than modern health care providers. Second, the healer offers much cheaper treatments and medications [52].

## DISCUSSION

### Main findings

The main findings of our review were threefold: First, we identified vulnerable populations by measuring disadvantages across social categories by means of the PROGRESS categories (“Who are the disadvantaged populations?”). Second, we tried to identify the geographies of such disadvantaged groups (“Where do they live?”). And third, we wanted to answer why and how their inequitable distribution of health is explained in terms of the SDH framework.

#### Who?

In this review we filtered out specific disadvantaged groups of children throughout the Indonesian archipelago who are in need for actionable health policy recommendations. Children living in remote rural communities are facing greater challenges to survive their fifth birthday compared to their urban counterparts. Children from low-income households and those who have less educated parents likewise are at a higher risk for negative health outcomes and disease development. We found that the ethnicity and the gender of the child do not play a critical role in Indonesia. However, the role of the mother and foremost her ability to accumulate human and social capital and engage in social or community participation had great impact on child health outcomes. These inequity barriers are not restricted to the Indonesian setting but also jeopardize the MDG 4 achievement of other countries in Southeast Asia. For instance, while socioeconomic position remains as a strong determinant of child health in Indonesia, others studies showed that e.g. Vietnam has successfully targeted disadvantaged socioeconomic groups and lessened the impact of socioeconomic position as a main determinant of child health through equitable national programs. However, ethnicity remained a strong determinant of poor child health in Vietnam due to prevailing discrimination and marginalization of ethnic minorities [8, 102]. We found that the gender of the child is almost neglectable when it comes to child health outcomes in Indonesia. The opposite can be said for countries like China [103] or India [104] where gender differences in child survival remain rather pronounced. Results like such describe horizontal inequities (= inter-group inequities; e.g. disparities between groups of different levels of socioeconomic status, gender or ethnicity) rather than vertical inequities (= inter-personal inequities; e.g. disparities between individuals across groups; e.g. geographic regions) [105]. Inter-group disparities account for the major inequalities in many countries and “closing this gap” has been a prime goal of the Commission on the Social Determinants of Health [106]. Monitoring and acting upon horizontal inequities—the kind of inequity that is created in the “tails” (e.g. between the richest and poorest segments of society) is crucial for policy makers addressing poverty reduction and aiming for an universal achievement of MDG 4 [107].

#### Where?

Our review found that place of residence (a potential source of vertical inequities) stems out as a strong contextual determinant of child health. Indonesia is comprised of 33 provinces with 497 districts and 54% of all children live in urban areas. The latest MDG achievement report stated that the country will successfully reduce its under-five mortality rates following a seemingly linear decline from 97 to 32 deaths per 1,000 life births from 1990 to 2015 [7]. However, as pointed out before, there are huge inter-province and rural—urban discrepancies resulting in 27 provinces having higher infant mortality rates than the national average. Further, under-five mortality rates in the Yogyakarta Special Region (22/1,000) is almost three times lower than in West Sulawesi (96/1,000). According to data from the Indonesian Demographic Health Surveys from 1997 and 2007, the rural infant mortality rates dropped from 58 to 45 but remain high compared to the urban rates, which declined from 35 to 31 deaths per 1,000 life births [108]. Equitable progress towards the MDG 4 targets implies that the health outcomes of the most disadvantaged (e.g. in rural areas) will improve at least at the same rates as the better-off groups (e.g. in urbanized provinces). The second aim of our review was to identify the geographies of disadvantaged groups in terms of child health. Similar to the first aim, we were unable to track and identify in detail the specific regional variation of disadvantaged groups. The main reason for this stems from the fact that the majority of the included studies in this review made use of publicly available secondary survey data such as the Indonesian Family Life Survey (IFLS), the Indonesian Demographic Health Survey (IDHS), the Nutrition and Health Surveillance System (NSS), the Indonesia Demographic and Health Survey or datasets from BAPPENAS (Statistics Indonesia). However, one of the constraints with survey data is that it leaves out those parts of the population that are the hardest to reach (i.e. the poorest in remote areas) and rather provides the “average” or “mean” health in the population. Statistics on equity in health are rare because only few studies facilitate this data in a way that analyses are broken down to anything smaller than sub-provincial level. In order to monitor MDG 4 in an equity-oriented (as opposed to an “equity-blind”) and adjusted manner it is necessary to stratify the population under study by rural/urban residence, income, educational status, or by other structural determinants of health, which can yield child health disparities across numerous health indicators [109]. Besides requests for rigorous analysis of mortality trends, there is also a clearly expressed need for improved health metrics among the research community. According to Reidpath et al., a composite indicator that combines the rates as well as the social distributions of under-five mortality would contribute greatly to a reorientation of the global health agenda [5]. It is obvious that different sub-populations suffer disproportionally and thus the recorded MDG success is not universal and needs to be interpreted carefully. This lack of SDH disaggregated data results in the fact that many groups will continue to fall out of the radar of appropriate interventions [110]. This review adds to the pool of research that a countries’ performance needs to be judged at sub-national level to make sure that marginalized and vulnerable populations are not suffering from an unequal and inequitable distribution of power, money and resources. Even though we could not identify in detail where disadvantaged populations reside across the Indonesian archipelago, we were able to shed light on the fact that MDG 4 does not decline in a purely linear manner and that especially geographical disparities in child health are disguised in and by research. It is crucial for global public health beyond 2015 to tackle the shortfalls of the MDGs—especially their quantification of equity that enhances health inequity among the most disadvantaged. At this stage it remains open in how far the Sustainable Development Goals (SDGs)—the MDG replacement system after 2015, will fulfill such expectations.

#### Why and how?

Our last aim was to answer why and how this inequitable distribution of health is explained in terms of the social determinants of health model. Our review presented various mechanisms on how structural determinants work through a range of intermediary factors and impact on child health (Fig 1). Since it is beyond the scope of this paper to discuss each mechanism in detail we focus on the five most prominent pathways, which have been defined throughout our review:

*(i) Rural health systems and child health care*. Globally, parents in rural areas face difficulties in accessing high quality health care services for their children. Such disadvantage results in increased morbidity and mortality rates. For instance, the ratio of rural to urban under-five mortality is 1.6 in most LMICs and rural-urban disparities are even more pronounced in the South-East Asia region where rural children are 1.8 times more likely not to survive their fifth birthday compared to their urban counterparts [111]. Our review has substantiated this pattern of wide rural-urban disparities in under-five, infant and neonatal mortality in Indonesia. The rural health care system is the most well researched intermediate determinant of child health inequities in Indonesia. We mapped out manifold associations between child health and access and availability of health centers, the quality of services, and the utilization of care in rural Indonesian settings. In a country where almost sixty per cent of the population resides in rural places across a variety of heterogeneous geographies, it is of utmost importance that the relationship between place of residence and child health is fully understood in order to tackle persistent disparities in child health outcomes. In accordance with our findings, geographical access to health facilities was found to be associated with higher neonatal and child mortality also in other Asian (for example rural northern Vietnam [112]) and African (for example remote Ethiopia [113]) settings. However, one study on the influence of distance to delivery care on early neonatal mortality in Malawi and Zambia could not identify that better geographic access improves early neonatal health [114], confirming the findings from our review stating that very small health benefits derive from increased availability of health services when quality of care does not improve in rural settings. In Indonesia, the majority of people rely heavily on the internal and structural quality and infrastructure of rural health care systems. However, in many instances the rural health care system is not a determinant of child health *per se* but rather mediates ancillary effects of other structural determinants such as education and income or gender and ethnicity.

*(ii) Food poverty and child malnutrition*. Despite considerable economic growth throughout the last decades, still in 2014, 28 million Indonesians (11% of the population) currently live below the poverty line and approximately half of all households cluster around the national poverty line set at 200,262 IDR per month (USD 22). Thus, income and economic status have continued to be the main structural determinants of child health inequities in Indonesia. Our review stressed the important impact of materialistic risk factors on child malnutrition and subsequent child mortality in Indonesia. We have shown that access and quality of food strongly depends on households’ purchasing power. Our results are in line with a study by Singh et al. who identified food poverty as main socioeconomic determinant of child health in India [115] or Malqvist et al. who found similar patterns for the Vietnamese context [116] and a cross-country report on child malnutrition and mortality which identified main clusters of food insecure and vulnerable households in different LMICs [117]. The gap between rich and poor is still widening in Indonesia and children from low-income families are disadvantaged in many ways, which calls for targeted policy actions. A review about the effectiveness of interventions to reduce inequities in child and maternal health in LMICs specifically calls for nutrition supplement programs (such as Vitamin A, micronutrient and iron/folic acid supplementation) to reduce child and neonatal mortality among socioeconomically disadvantaged groups [118]. Indonesia is currently undergoing a nutrition transition due to its fast economic development and urbanization. Food patterns shift towards what is often called a “western diet” that is rich in refined carbohydrates, sugars and fats [119]. Our review has shown that mothers entering the labor market tend to breastfeed their children less and shorter. Breastfeeding has been shown to having both an immediate health protective effect during infancy but also during the remaining life course. In this sense, two rising phenomenon are worth mentioning: the rise of a dual burden of malnutrition (the presence of both overweight and underweight) which currently affects 19% of all Indonesian households [120] and the epidemiological transition towards a rise of chronic non-communicable diseases [121] which currently cause 64% of all deaths in Indonesia [122]. Our review has not identified overweight/obesity or chronic diseases such as diabetes as serious health concerns among Indonesian children. However, there are indications that these will be the problems of the generations to come as the country continuous to experience health and economic transitions.

*(iii) Health literacy and child health outcomes*. It is widely acknowledged that education mediates health through different pathways, e.g. by increasing health literacy and promoting healthy behaviors or by shaping employment and income opportunities. Even though maternal education has long been considered as one of the most crucial determinants of child health, both maternal and paternal education are strongly associated with protective childcare behaviors, e.g. education enables parents to access health care services and to communicate with health staff. One of the prominent findings of this review were the associations between parents’ health literacy and child health promotion in terms of increased access to health care and higher uptake of immunization or supplementation services, optimal feeding practices and an decreased exposure to environmental tobacco smoke. These findings are in line with a review on health literacy and child health outcomes stating that parents with low literacy showed behaviors that were less advantageous for their children's health compared to parents with higher health literacy. However, the same authors found mixed results for the relationship of literacy to the use of health care services [123]. Sanders et al. linked poor health literacy with uptake of poor preventive care behaviors and calls out for future research and interventions aiming at ameliorate literacy-associated child health disparities [124].

Inequalities perpetuated by health care workers being selective in their provision of advice and services seriously jeopardizes the continuum of care and puts children at greater risk of unmet medical needs. Our review mapped out examples of socioeconomic discrimination in health care provision based on parental income and educational attainment. This is consistent with findings from other studies conducted in similar socioeconomic settings. For instance, Singh et al. in two studies on the provision of public health workers’ advice during antenatal care [125] and the use of postnatal care [115] in rural India found that the Indian health system was inclined to treat patients based on their socioeconomic background. For example, health care workers were increasingly biased in favor of higher income and higher educated patients leading to more utilization of antenatal, natal and postnatal care among the rich compared to socioeconomically disadvantaged households.

*(iv) Household decision-making and child health status*. Indonesia is a Muslim dominated country. Patriarchal and matriarchal structures are both common. This makes Indonesia an exceptional case compared to other countries in the South-East Asia Region. Our review clearly showed that there are vast research and knowledge gaps regarding gender and child health in Indonesia. Still, our main results in this area were twofold: first, we could not identify a specific son preference among Indonesian parents or vice versa we could not identify an issue of “missing daughters”. A global review on gender bias in pediatric healthcare stated that most studies emerge from Asian and Southeast Asian countries such as Vietnam [126], China [103, 127] or India [104] and only sporadically from Africa or South America [128]. Second, which is in line with the first result, we were able to explore pathways of mothers’ household decision making (expressed for example in her bargaining power in marriage and her community participation) on child health status. These findings are in line with a study from Bangladesh claiming that economic up-lift at family level was more likely to positively affect female empowerment and increased health care service utilization [129]. This was also in line with studies claiming that gender roles influence the perception of illness but not care seeking *per se* as shown in a study from Nepal [130] or with studies in other South-East Asia countries reporting a lack of decision making power and subsequent negative impacts on child health [116]. A recent study investigated households suffering from a dual burden of malnutrition emphasizes the importance of future women empowerment for reducing social, nutritional and health inequalities in Indonesia [120]. Although we could not identify gender (of the child) as a strong determinant of health, it remains a fact that Indonesia currently ranks 95^th^ in the 2013 World Gender Gap Report indicating that the country has some way to go to successfully bridge gender gap in many areas including health [131].

*(v) Traditional birth attendants and child health behaviors*. More than 300 ethnic minority groups reside on the Indonesian archipelago. The overall situation described in our review is that ethnic minority groups are worse off in terms of child health. Major contributing factors were low accessibility and affordability of modern health care services (antenatal, perinatal and postnatal care services) and the preferred use of a traditional healer compared to facility delivery. The World Health Organization defines traditional birth attendants (TBAs) as laypersons assisting a mother during pregnancy and childbirth. The role of the TBAs varies according to geographical and cultural aspects. However, TBAs are commonly highly respected older women who may either work independently or in collaboration with a facility within the health care system. TBA services were temporally and spatially easily available, lower charged and more cultural sensitive [132]. Our review was able to confirm this for the Indonesian context. A review on ethnic minority health in Vietnam identified health care seeking behavior, utilization of maternal and child health services and nutrition as main areas along which ethnic health inequities evolve [133]. A review on disadvantaged populations in China likewise identified ethnicity as a strong determinant for insufficient knowledge of reproductive health matters, reduced rates of facility based deliveries and poor maternal health in general [134]. In many LMICs, traditional birth attendants provide most of maternity and new-born care attributing them a prominent role in maternal, neonatal and child health [135]. Several studies identified that the key missing piece to improve the health of children delivered by a TBA is to introduce a referral system linking TBAs to skilled midwifes [136] or to design and implement effective behavior change communication strategies to overcome cultural or religious barriers that prevents mothers from seeking care from TBAs [137]. We could not accumulate much evidence for a direct ethnicity-based discrimination and child health care provision in Indonesia. In fact, in many other country settings, the quality and amount of healthcare received by ethnic minorities and women is closely linked to economic inequalities and less to mere ethnic belonging or gender-based discrimination. A study by Brockenhoff and Hewett comparing child health inequalities among different ethnic groups in eleven sub-Saharan countries concluded that ethnic mortality differences are closely linked with economic inequalities [138]. Burgard and Treiman likewise conclude that the ethnic gradient in child mortality among South African Blacks and Whites was mainly explained by household economic status and parental education [139]. Many studies considered ethnicity as a covariate of low income or remote place of residence. More research is needed to fully understand ethnicity as a determinant of child health in Indonesia.

### Strengths and limitations

Our systematic review comprises several key strengths. The search strategy has been comprehensive and covers three contentually different databases ranging from biomedical and reproductive health perspectives to the social sciences. The inclusion and exclusion criteria were well defined and carefully applied to each study and the systematic review as a whole evolved along the principal guidelines of PRISMA-Equity and the SDH framework. In our opinion it is the first published systematic review on child health inequities in Indonesia and we believe that this brings forward novel insight into a topic which must receive utmost priority during the short time until December 2015. We did not only map the current situation of MDG 4 with respect to disadvantaged populations in Indonesia applying the SDH framework but we also present research gaps and potential working points for policy interventions. By this we hope to provide inputs for the post-2015 developing goals in Indonesia and similar developing settings.

There are also limitations associated with the conduct of our systematic literature review. We omitted studies published in languages other than English; also no publications in Indonesian were included. However, we assume that the impact of language bias is rather low. It has been noted by other authors that the exclusion of studies reported in a language different than English did not significantly affect the results of individual systematic reviews and meta-analyses [140, 141]. Further, we did not exclude studies based on small geographical coverage nor their methodological quality. The reason for this is that we intend to map out the overall situation and causes of child health inequities in Indonesia and not to measure the magnitude of inequity *per se*. Further, we *a priori* excluded experimental studies or clinical trials and thus do not consider any interventions reducing child health inequities in Indonesia-even if proven to be efficient. This, to a certain extent, limits the picture of child health inequities since it only focuses on the problem and not on what has been achieved to curb health inequities. Conducting a systematic literature review on effective interventions in child health in Indonesia could be a future research address. Secondly, the majority of the studies included did not evolve along an elaborated conceptual framework of inequality but rather stratify their outcomes of interest by variables such as education, income or ethnicity. Such a limitation has already been recognized by Målqvist et al. who conducted a qualitative literature review on maternal and child health in Vietnam [116]. The absence of a conceptual framework—such as the SDH hinders many individual studies to have a holistic view on at the pathways that create or detain child health inequities. Therefore, a key strength of our systematic review is that we apply the SDH framework to many studies that lack such a viewpoint. Studies applying the SDH framework by arranging their variables in a hierarchical way so that it can be shown how structural determinants work though a set of intermediary determinants and eventually influence child health is clearly a research gap. Moreover, longitudinal analyses and studies on how certain relationships (e.g. parents’ health literacy and child health) are mediated as well as more advanced analytical qualitative approaches to child health inequities (e.g. Grounded theory) likewise remain as research gaps. While we were able to elaborate on the conceptualizations and inferences about “why and how” child health inequities exist and persist across the Indonesian archipelago, a limitation derives from the fact that were we were less able to track and identify in such detail “who” are the disadvantaged groups in the community and to track their specific regional variation (“where?”). As we have discussed above, such a limitation derives from the nature of national surveys on which most included studies of this review (as well as the assessment of the MDG achievements itself) rely. We acknowledge this limitation and prospect that this will add to the on-going discussions about how to conceptualize and measure health inequity, design and scale-up high-impact intervention, and how to measure intervention coverage beyond 2015 [5, 142, 143].

### Policy implications

The route from evidence to policy to practice is a complex interaction between science, policy and politics. The end of 2015 deadline for achieving MDG 4 is approaching and the notion of health equity and the social determinants of health have gained momentum. What has become obvious through the last decades is that the major impediment to tackle social determinants of child health inequities is not a lack of evidence (although there is) but rather a lack of firm political commitment to it [144]. Working strategies to accelerate and improve progress to achieve child health among disadvantaged populations require need-oriented and context-sensitive actions with defined short, medium and long-term goals. This review identified five key determinants of poor child health in Indonesia and our work has also drawn attention to the fact that there remains great need for a more elaborated evidence base on health inequities, social determinants of health and child health interventions.

In their 2010 and 2011 MDG Achievement Reports, the Indonesian government has outlined persisting challenges and efforts to improve child health [7, 30]. “Table 6” intends to summarize this evidence and provide actionable policy recommendations for the priority groups and the five key determinants identified in our review. Our recommendations are structured according to the three principles on the social determinants of health [106]: (i) improving daily living conditions; (ii) tackle the inequitable distribution of power, money and resources and (iii) measure and understand the problem and assess the results of action.

**Table 6:** Short, medium, and long-term policies and efforts to accelerate improvements along the five key determinants of child health inequities and for closing the gap in child health in Indonesia.

	Short-term Perspective: *Improve daily living conditions*	Medium-term Perspective: *Tackle inequitable distribution of power*, *money and resources*	Long-term Perspective: *Measure and understand the problem and assess the impact of action*
**Child Health Policies**	Improving measles immunization coverage; Strengthening key IMCI implementations; Addressing key nutritional concerns to reduce stunting; Develop family-level child health strategies; Strengthen behavior change interventions at household level; Improving new-born care and maternal health	Strengthen and improve health care facilities; Improve community participation and mobilization; Enhance policy advocacy for disadvantaged provinces	Integrate cross-sectoral strategies to accelerate achievements of target for child, infant, and neonatal mortality
**Gaps and Recommendations**	Comprehensive approach to early life development; Socially cohesive communities and neighborhoods; Better urban planning; Address exclusionary policies and processes that lead to rural poverty; Ensure fair employment and decent working conditions, reduce exposure to physical and psychosocial hazards; Universal social protection system and publicly funded health-care system based in primary health care with minimum out-of-pocket spending; Investment in training and retaining health care workers	Intersectoral coordinated action on health and policy coherence; *Finance*: strengthened, coherent and cross-sectoral public financing to improve SDH, progressive taxation and fair allocation; greater poverty reduction and debt relief; *Markets*: public-sector leadership for effective regulation of products, activities and conditions that damage health; regular health equity impact assessment of all market regulation policies; Promote *gender equity*; *Political empowerment* across various intersecting social categories especially ethnic minorities	Data which does not mask health disparities; Elaborated evidence base on health inequity, SDH, and interventions; Capacity building among policy makers, practitioners and other stakeholders; Routine monitoring systems

Our findings also attach great importance to the understanding of the interrelationships between MDG 4 and the other seven goals. As others have already brought forward, the eight MDGs should not be regarded or achieved as independent bundles but as a dynamic process [145]. For instance, our review has shown that in Indonesia the reduction of child mortality (MDG 4) does highly depend on the eradication of poverty and hunger (MDG 1), the promotion of universal education (MDG 2) as well as gender equality and women’s empowerment (MDG 3). In addition, reducing maternal mortality (MDG 5) presents a key point of intersection—foremost for a reduction in neonatal mortality. National MDG statistics show that the country is facing problems with achieving MDG 1 and MDG 5 [7, 30]. For Indonesia, very little is known about how much progress on MDG 5 impacts on the achievement of MDG4 since multi-goal or cross-goal analyses are rare. In order to successfully reduce child mortality, any policy actions or investments must be tailored to reach the populations at highest risk.

## Conclusion

Indonesia is a highly populated and heterogeneous place and the health of its children is challenged by the country’s geographic, demographic, political, socioeconomic, and cultural diversity in many aspects. However, even under such challenging circumstances it remains almost a universal fact that the majority of deaths among children under the age of five can be prevented. The progress towards MDG 4 in Indonesia is not universal as well is this goals equity-blind and obscures health disparities within different population groups. This review shows that the major determinants of poor child health in Indonesia are subject to the shortcomings of the health system, malnutrition, low health literacy, as well as gender and ethnic inequalities. This work reviews existing evidences and calls for enhanced understanding of the determinants and pathways that produce and overcome inequities in health among a disadvantaged and vulnerable population such as children.

## Supporting Information

S1 PRISMA Checklist (PDF)

S1 Search Syntax (PDF)

## References

[1] United Nations General Assembly. United Nations Millennium Declaration: Resolution adopted by the General Assembly. 55/2 18 9 2000, 2000, United Nations: New York.

[2] United Nations. Millennium Development Goals report 2013, 2013, United Nations: New York.

[3] Wang H, Liddell CA, Coates MM, Mooney MD, Levitz CE, Schumacher AE, et al Global, regional, and national levels of neonatal, infant, and under-5 mortality during 1990–2013: a systematic analysis for the Global Burden of Disease Study 2013. Lancet. 2014. 10.1016/S0140-6736(14)60497-9 PMC4165626 24797572

[4] United Nations. The Millennium Development Goals Report 2014, 2014, United Nations: New York

[5] Reidpath DD, Morel CM, Mecaskey JW, Allotey P. The Millennium Development Goals fail poor children: the case for equity-adjusted measures. PLoS Med. 2009; 6(4): p. e1000062 10.1371/journal.pmed.1000062 19399155 PMC2667271

[6] UNICEF. Progress for children: achieving the MDGs with equity, 2010, UNICEF: New York.

[7] Ministry of National Development Planning and National Development Planning Agency (BAPPENAS). Report on the achievements of the Millennium Development Goals in Indonesia 2011, 2012, BAPPENAS: Jakarta.

[8] Thomsen S, Hoa DT, Malqvist M, Sanneving L, Saxena D, Tana S, et al Promoting equity to achieve maternal and child health. Reprod Health Matters. 2011; 19(38): p. 176–182. 10.1016/S0968-8080(11)38586-2 22118151

[9] Welch V, Petticrew M, Tugwell P, Moher D, O'Neill J, Waters E, et al PRISMA-Equity 2012 extension: reporting guidelines for systematic reviews with a focus on health equity. PLoS Med. 2012; 9(10): p. e1001333 10.1371/journal.pmed.1001333 23222917 PMC3484052

[10] Thomsen S, Ng N, Biao X, Bondjers G, Kusnanto H, Liem NT, et al Bringing evidence to policy to achieve health-related MDGs for all: justification and design of the EPI-4 project in China, India, Indonesia, and Vietnam. Glob Health Action. 2013; 6. 10.3402/gha.v6i0.19650 PMC3597775 23490302

[11] Culyer AJ. Equity—some theory and its policy implications. J Med Ethics. 2001; 27: p. 275–283. 11479360 10.1136/jme.27.4.275 PMC1733434

[12] Sen A. Why health equity? Health Econ. 2002; 11(8): p. 659–666. 12457367 10.1002/hec.762

[13] Commission on Social Determinants of Health. Closing the gap in a generation: health equity through action on the Social Determinants of Health, 2008, World Health Organization: Geneva. 10.1016/S0140-6736(08)61690-6 18994664

[14] Friel S, Marmot MG. Action on the social determinants of health and health inequities goes global. Annu Rev Public Health. 2011; 32: p. 225–236. 10.1146/annurev-publhealth-031210-101220 21219162

[15] Tugwell P, Petticrew M, Kristjansson E, Welch V, Ueffing E, Waters E, et al Assessing equity in systematic reviews: realising the recommendations of the Commission on Social Determinants of Health. BMJ. 2010; 341: p. c4739 10.1136/bmj.c4739 20837575

[16] Hsieh HF, Shannon SE. Three approaches to qualitative content analysis. Qual Health Res. 2005; 15(9): p. 1277–1288. 16204405 10.1177/1049732305276687

[17] Krippendorff K. Content analysis: an introduction to its methodology 2004, Thousand Oaks: SAGE.

[18] Dixon-Woods M, Agarwal S, Jones D, Young B, Sutton A. Synthesising qualitative and quantitative evidence: a review of possible methods. J Health Serv Res Policy. 2005; 10(1): p. 45–53. 15667704 10.1177/135581960501000110

[19] Mellington N, Cameron L. Female education and child mortality in Indonesia. Bulletin of Indonesian Economic Studies. 1999; 35(3): p. 115–144. 12349697 10.1080/00074919912331337717

[20] Semba RD, Kraemer K, Sun K, de Pee S, Akhter N, Moench-Pfanner R, et al Relationship of the presence of a household improved latrine with diarrhea and under-five child mortality in Indonesia. American Journal of Tropical Medicine and Hygiene. 2011; 84(3): p. 443–450. 10.4269/ajtmh.2011.10-0244 21363984 PMC3042822

[21] Paknawin-Mock J, Jarvis L, Jahari AB, Husaini MA, Pollitt E. Community-level determinants of child growth in an Indonesian tea plantation. Eur J Clin Nutr. 2000; 54 Suppl 2: p. S28–42. 10902985 10.1038/sj.ejcn.1601003

[22] BPS (Statistics Indonesia). Multiple indicator cluster survey on the education and health of mothers and children, 2000, Statistics Indonesia: Jakarta p. viii, 52 p.

[23] Sari M, de Pee S, Bloem MW, Sun K, Thorne-Lyman AL, Moench-Pfanner R, et al Higher household expenditure on animal-source and nongrain foods lowers the risk of stunting among children 0–59 months old in Indonesia: implications of rising food prices. J Nutr. 2010; 140(1): p. 195S–200S. 10.3945/jn.109.110858 19939994

[24] Semba RD, de Pee S, Sun K, Sari M, Akhter N, Bloem MW. Effect of parental formal education on risk of child stunting in Indonesia and Bangladesh: a cross-sectional study. Lancet. 2008; 371(9609): p. 322–328. 10.1016/S0140-6736(08)60169-5 18294999

[25] Ng CS, Dibley MJ, Agho KE. Complementary feeding indicators and determinants of poor feeding practices in Indonesia: a secondary analysis of 2007 Demographic and Health Survey data. Public Health Nutr. 2012; 15(5): p. 827–839. 10.1017/S1368980011002485 22014663

[26] Helen Keller International. High prevalence of anemia among young children in urban and rural areas. HKI Indonesia Crisis Bulletin. 2000; 2(1): p. 1–4.

[27] Doak CM, Adair LS, Bentley M, Monteiro C, Popkin BM. The dual burden household and the nutrition transition paradox. Int J Obes. 2005; 29(1): p. 129–136. 15505634 10.1038/sj.ijo.0802824

[28] Oddo VM, Rah JH, Semba RD, Sun K, Akhter N, Sari M, et al Predictors of maternal and child double burden of malnutrition in rural Indonesia and Bangladesh. Am J Clin Nutr. 2012; 95(4): p. 951–958. 10.3945/ajcn.111.026070 22357721

[29] Ministry of National Development Planning and National Development Planning Agency (Bappenas). Indonesia progress report on the Millenium Development Goals, G.o.I. Bappenas, Editor 2004: Jakarta.

[30] Ministry of National Development Planning and National Development Planning Agency (Bappenas). Report on the achievements of the Millennium Development Goals Indonesia 2010, 2010, Bappenas, Government of Indonesia Jakarta.

[31] Fernandez R, Rammohan A, Awofeso N. Correlates of first dose of measles vaccination delivery and uptake in Indonesia. Asian Pac J Trop Med. 2011; 4(2): p. 140–145. 10.1016/S1995-7645(11)60055-2 21771439

[32] Fernandez RC, Awofeso N, Rammohan A. Determinants of apparent rural-urban differentials in measles vaccination uptake in Indonesia. Rural Remote Health. 2011; 11(3): p. 1702 21899375

[33] Houweling TA, Kunst AE, Borsboom G, Mackenbach JP. Mortality inequalities in times of economic growth: time trends in socioeconomic and regional inequalities in under 5 mortality in Indonesia, 1982–1997. J Epidemiol Community Health. 2006; 60(1): p. 62–68. 16361456 10.1136/jech.2005.036079 PMC2465535

[34] Nelson CM, Sutanto A, Gessner BD, Suradana IG, Steinhoff MC, Arjoso S. Age- and cause-specific childhood mortality in Lombok, Indonesia, as a factor for determining the appropriateness of introducing Haemophilus influenzae type b and pneumococcal vaccines. J Health Popul Nutr. 2000; 18(3): p. 131–138. 11262765

[35] Serquina-Ramiro L, Kasniyah N, Inthusoma T, Higginbotham N, Streiner D, Nichter M, et al Measles immunization acceptance in Southeast Asia: past patterns and future challenges. Southeast Asian J Trop Med Public Health. 2001; 32(4): p. 791–804. 12041556

[36] Dibley MJ, Titaley CR, d'Este C, Agho K. Iron and folic acid supplements in pregnancy improve child survival in Indonesia. Am J Clin Nutr. 2012; 95(1): p. 220–230. 10.3945/ajcn.111.022699 22170356

[37] Titaley CR, Dibley MJ, Roberts CL, Hall J, Agho K. Iron and folic acid supplements and reduced early neonatal deaths in Indonesia. Bull World Health Organ. 2010; 88(7): p. 500–508. 10.2471/BLT.09.065813 20616969 PMC2897981

[38] Senarath U, Dibley MJ, Agho KE. Factors associated with nonexclusive breastfeeding in 5 east and southeast Asian countries: a multilevel analysis. J Hum Lact. 2010; 26(3): p. 248–257. 10.1177/0890334409357562 20110564

[39] de Pee S, Moench-Pfanner R, Bloem MW. Insight into breastfeeding and complementary feeding practices: a case study from Indonesia. SCN NEWS. 2003(27): p. 23–27.

[40] Dijkhuizen MA, Wieringa FT, West CE, Muherdiyantiningsih, Muhilal. Concurrent micronutrient deficiencies in lactating mothers and their infants in Indonesia. Am J Clin Nutr. 2001; 73(4): p. 786–791. 11273854 10.1093/ajcn/73.4.786

[41] Ministry of National Development Planning and National Development Planning Agency (Bappenas). Report on the achievements of the Millennium Development Goals Indonesia 2007, 2007, Bappenas, Government of Indonesia Jakarta.

[42] Berger SG, de Pee S, Bloem MW, Halati S, Semba RD. Malnutrition and morbidity are higher in children who are missed by periodic vitamin A capsule distribution for child survival in rural Indonesia. J Nutr. 2007; 137(5): p. 1328–1333. 17449600 10.1093/jn/137.5.1328

[43] Berger SG, de Pee S, Bloem MW, Halati S, Semba RD. Malnutrition and morbidity among children not reached by the national vitamin A capsule programme in urban slum areas of Indonesia. Public Health. 2008; 122(4): p. 371–378. 10.1016/j.puhe.2007.08.003 18222504

[44] Titaley CR, Dibley MJ, Agho K, Roberts CL, Hall J. Determinants of neonatal mortality in Indonesia. BMC Public Health. 2008; 8: p. 232 10.1186/1471-2458-8-232 18613953 PMC2478684

[45] Titaley CR, Dibley MJ, Roberts CL. Factors associated with non-utilisation of postnatal care services in Indonesia. J Epidemiol Community Health. 2009; 63(10): p. 827–831. 10.1136/jech.2008.081604 19414443

[46] Titaley CR, Dibley MJ, Roberts CL. Factors associated with underutilization of antenatal care services in Indonesia: results of Indonesia Demographic and Health Survey 2002/2003 and 2007. BMC Public Health. 2010; 10: p. 485 10.1186/1471-2458-10-485 20712866 PMC2933719

[47] Titaley CR, Dibley MJ, Roberts CL. Utilization of village midwives and other trained delivery attendants for home deliveries in Indonesia: results of Indonesia Demographic and Health Survey 2002/2003 and 2007. Matern Child Health J. 2011; 15(8): p. 1400–1415. 10.1007/s10995-010-0697-1 20936501

[48] Titaley CR, Hunter CL, Dibley MJ, Heywood P. Why do some women still prefer traditional birth attendants and home delivery?: a qualitative study on delivery care services in West Java Province, Indonesia. BMC Pregnancy Childbirth. 2010; 10: p. 43 10.1186/1471-2393-10-43 20701762 PMC2928756

[49] Titaley CR, Hunter CL, Heywood P, Dibley MJ. Why don't some women attend antenatal and postnatal care services?: a qualitative study of community members' perspectives in Garut, Sukabumi and Ciamis districts of West Java Province, Indonesia. BMC Pregnancy Childbirth. 2010; 10: p. 61 10.1186/1471-2393-10-61 20937146 PMC2964562

[50] Barber SL, Gertler PJ, Harimurti P. The contribution of human resources for health to the quality of care in Indonesia. Health Aff. 2007; 26(3): p. w367–379. 17389634 10.1377/hlthaff.26.3.w367

[51] Barber SL, Gertler PJ, Harimurti P. Differences in access to high-quality outpatient care in Indonesia. Health Aff. 2007; 26(3): p. w352–366. 17389632 10.1377/hlthaff.26.3.w352

[52] Grace J. The treatment of infants and young children suffering respiratory tract infection and diarrhoeal disease in a rural community in Southeast Indonesia. Soc Sci Med. 1998; 46(10): p. 1291–1302. 9665561 10.1016/s0277-9536(97)10057-0

[53] Thind A. Analysis of health services use for respiratory illness in Indonesian children: implications for policy. J Biosoc Sci. 2005; 37: p. 129–142. 15768769 10.1017/s002193200300645x

[54] Thind A, Banerjee K. Home deliveries in Indonesia: who provides assistance? J Community Health. 2004; 29(4): p. 285–303. 15186015 10.1023/b:johe.0000025327.70959.d3

[55] Semba RD, de Pee S, Berger SG, Martini E, Ricks MO, Bloem MW. Malnutrition and infectious disease morbidity among children missed by the childhood immunization program in Indonesia. Southeast Asian J Trop Med Public Health. 2007; 38(1): p. 120–129. 17539257

[56] Frankenberg E, Suriastini W, Thomas D. Can expanding access to basic healthcare improve children's health status? Lessons from Indonesia's 'midwife in the village' programme. Popul Stud (Camb). 2005; 59(1): p. 5–19. 15764131 10.1080/0032472052000332674

[57] Osaki K, Hattori T, Kosen S. The role of home-based records in the establishment of a continuum of care for mothers, newborns, and children in Indonesia. Glob Health Action. 2013; 6: p. 1–12. 10.3402/gha.v6i0.20429 PMC3647040 23651873

[58] Bose-O'Reilly S, Lettmeier B, Roider G, Siebert U, Drasch G. Mercury in breast milk: a health hazard for infants in gold mining areas? Int J Hyg Environ Health. 2008; 211(5–6): p. 615–623. 18262466 10.1016/j.ijheh.2007.09.015

[59] Toyama N, Wakai S, Nakamura Y, Arifin A. Mother's working status and nutritional status of children under the age of 5 in urban low-income community, Surabaya, Indonesia. J Trop Pediatr. 2001; 47(3): p. 179–181. 11419684 10.1093/tropej/47.3.179

[60] Ramli, Agho KE, Inder KJ, Bowe SJ, Jacobs J, Dibley MJ. Prevalence and risk factors for stunting and severe stunting among under-fives in North Maluku province of Indonesia. BMC Pediatr. 2009; 9: p. 64 10.1186/1471-2431-9-64 19818167 PMC2765943

[61] Waters H, Saadah F, Surbakti S, Heywood P. Weight-for-age malnutrition in Indonesian children, 1992–1999. Int J Epidemiol. 2004; 33(3): p. 589–595. 15155707 10.1093/ije/dyh074

[62] Block SA, Kiess L, Webb P, Kosen S, Moench-Pfanner R, Bloem MW, et al Macro shocks and micro outcomes: child nutrition during Indonesia's crisis. Econ Hum Biol. 2004; 2(1): p. 21–44. 15463991 10.1016/j.ehb.2003.12.007

[63] de Pee S, Bloem MW, Sari M, Kiess L, Yip R, Kosen S. The high prevalence of low hemoglobin concentration among Indonesian infants aged 3–5 months is related to maternal anemia. J Nutr. 2002; 132(8): p. 2215–2221. 12163665 10.1093/jn/132.8.2215

[64] Helen Keller International. Decreasing prevalence of anemia among urban children: Does it indicate increased access to micronutrient-rich foods? HKI Indonesia Crisis Bulletin. 2000; 2(3): p. 1–4.

[65] Campbell AA, Thorne-Lyman A, Sun K, de Pee S, Kraemer K, Moench-Pfanner R, et al Greater household expenditures on fruits and vegetables but not animal source foods are associated with decreased risk of under-five child mortality among families in rural Indonesia. J Nutr. 2008; 138(11): p. 2244–2249. 18936226 10.1093/jn/138.11.2244

[66] Semba RD, de Pee S, Hess SY, Sun K, Sari M, Bloem MW. Child malnutrition and mortality among families not utilizing adequately iodized salt in Indonesia. Am J Clin Nutr. 2008; 87(2): p. 438–444. 18258636 10.1093/ajcn/87.2.438

[67] Semba RD, Moench-Pfanner R, Sun K, de Pee S, Akhter N, Rah JH, et al Iron-fortified milk and noodle consumption is associated with lower risk of anemia among children aged 6–59 mo in Indonesia. Am J Clin Nutr. 2010; 92(1): p. 170–176. 10.3945/ajcn.2010.29254 20444956

[68] Semba RD, Moench-Pfanner R, Sun K, de Pee S, Akhter N, Rah JH, et al Consumption of micronutrient-fortified milk and noodles is associated with lower risk of stunting in preschool-aged children in Indonesia. Food Nutr Bull. 2011; 32(4): p. 347–353. 22590968 10.1177/156482651103200406

[69] Semba RD, de Pee S, Kraemer K, Sun K, Thorne-Lyman A, Moench-Pfanner R, et al Purchase of drinking water is associated with increased child morbidity and mortality among urban slum-dwelling families in Indonesia. Int J Hyg Environ Health. 2009; 212(4): p. 387–397. 10.1016/j.ijheh.2008.09.001 18976955

[70] Bardosono S, Sastroamidjojo S, Lukito W. Determinants of child malnutrition during the 1999 economic crisis in selected poor areas of Indonesia. Asia Pac J Clin Nutr. 2007; 16(3): p. 512–526. 17704034

[71] Poerwanto S, Stevenson M, de Klerk N. Infant mortality and family welfare: policy implications for Indonesia. J Epidemiol Community Health. 2003; 57(7): p. 493–498. 12821691 10.1136/jech.57.7.493 PMC1732515

[72] Gryboski KL. Maternal and non-maternal time-allocation to infant care, and care during infant illness in rural Java, Indonesia. Soc Sci Med. 1996; 43(2): p. 209–219. 8844925 10.1016/0277-9536(95)00363-0

[73] Roshita A, Schubert E, Whittaker M. Child-care and feeding practices of urban middle class working and non-working Indonesian mothers: a qualitative study of the socio-economic and cultural environment. Matern Child Nutr. 2012; 8(3): p. 299–314. 10.1111/j.1740-8709.2011.00298.x 21342457 PMC6860562

[74] Roshita A, Schubert E, Whittaker M. Child feeding practices in families of working and nonworking mothers of Indonesian middle class urban families: what are the problems? Ecol Food Nutr. 2013; 52(4): p. 344–370. 10.1080/03670244.2012.707438 23802915

[75] Dibley MJ, Senarath U, Agho KE. Infant and young child feeding indicators across nine East and Southeast Asian countries: an analysis of National Survey Data 2000–2005. Public Health Nutr. 2010; 13(9): p. 1296–1303. 10.1017/S1368980010000844 20441662

[76] Idris NS, Sastroasmoro S, Hidayati F, Sapriani I, Suradi R, Grobbee DE, et al Exclusive breastfeeding plan of pregnant Southeast Asian women: what encourages them? Breastfeed Med. 2013; 8(3): p. 317–320. 10.1089/bfm.2012.0003 23057643

[77] Best CM, Sun K, de Pee S, Sari M, Bloem MW, Semba RD. Paternal smoking and increased risk of child malnutrition among families in rural Indonesia. Tob Control. 2008; 17(1): p. 38–45. 10.1136/tc.2007.020875 18218806

[78] Semba RD, Campbell AA, Sun K, de Pee S, Akhter N, Moench-Pfanner R, et al Paternal smoking is associated with greater food insecurity among poor families in rural Indonesia. Asia Pac J Clin Nutr. 2011; 20(4): p. 618–623. 22094848

[79] Semba RD, Kalm LM, de Pee S, Ricks MO, Sari M, Bloem MW. Paternal smoking is associated with increased risk of child malnutrition among poor urban families in Indonesia. Public Health Nutr. 2007; 10(1): p. 7–15. 17212837 10.1017/S136898000722292X

[80] Semba RD, de Pee S, Sun K, Best CM, Sari M, Bloem MW. Paternal smoking and increased risk of infant and under-5 child mortality in Indonesia. Am J Public Health. 2008; 98(10): p. 1824–1826. 10.2105/AJPH.2007.119289 18309124 PMC2636455

[81] Sidik NA, Lazuardi L, Agung FH, Pritasari K, Roespandi H, Setiawan T, et al Assessment of the quality of hospital care for children in Indonesia. Trop Med Int Health. 2013; 18(4): p. 407–415. 10.1111/tmi.12061 23336605

[82] Frankenberg E. The effects of access to health care on infant mortality in Indonesia. Health Transit Rev. 1995; 5(2): p. 143–163. 10159677

[83] Thind A. Analysis of health services use for respiratory illness in Indonesian children: implications for policy. J Biosoc Sci. 2005; 37(2): p. 129–142. 15768769 10.1017/s002193200300645x

[84] Hatt L, Stanton C, Ronsmans C, Makowiecka K, Adisasmita A. Did professional attendance at home births improve early neonatal survival in Indonesia? Health Policy Plan. 2009; 24(4): p. 270–278. 10.1093/heapol/czp012 19339543

[85] Mostert S, Gunawan S, van Dongen JA, van de Ven PM, Sitaresmi MN, Wolters EE, et al Health-care providers' perspectives on childhood cancer treatment in Manado, Indonesia. Psychooncology. 2013; 22(11): p. 2522–2528. 10.1002/pon.3314 23703746

[86] Mostert S, Sitaresmi MN, Gundy CM, Sutaryo, Veerman AJ. Influence of socioeconomic status on childhood acute lymphoblastic leukemia treatment in Indonesia. Pediatrics. 2006; 118(6): p. e1600–1606. 17074838 10.1542/peds.2005-3015

[87] Mostert S, Sitaresmi MN, Gundy CM, Veerman AJ. Attitude of health-care providers toward childhood leukemia patients with different socio-economic status. Pediatr Blood Cancer. 2008; 50(5): p. 1001–1005. 17676594 10.1002/pbc.21324

[88] Usfar AA, Iswarawanti DN, Davelyna D, Dillon D. Food and personal hygiene perceptions and practices among caregivers whose children have diarrhea: a qualitative study of urban mothers in Tangerang, Indonesia. J Nutr Educ Behav. 2010; 42(1): p. 33–40. 10.1016/j.jneb.2009.03.003 20129187

[89] MacDonald SE, Moralejo MN, Matthews MK. Maternal understanding of diarrhoea-related dehydration and its influence on ORS use in Indonesia. Asia Pac J Public Health. 2007; 19(1): p. 34–39. 17784657 10.1177/10105395070190010701

[90] Semba RD, de Pee S, Ricks MO, Sari M, Bloem M. Diarrhea and fever as risk factors for anemia among children under age five living in urban slum areas of Indonesia. Int J Infect Dis. 2008; 12: p. 62–70. 17629535 10.1016/j.ijid.2007.04.011

[91] Webb KE, Horton NJ, Katz DL. Parental IQ and cognitive development of malnourished Indonesian children. Eur J Clin Nutr. 2005; 59(4): p. 618–620. 15688080 10.1038/sj.ejcn.1602103

[92] Yanuarti HP, Rusmil K, Effendi SH. Environment as risk factors in delay development in premature, LBW and mild asphyxia children. Pediatr Int. 2014. 10.1111/ped.12333 24617923

[93] Harjaningrum AT, Kartasasmita C, Orne-Gliemann J, Jutand MA, Goujon N, Koeck JL. A qualitative study on knowledge, perceptions, and attitudes of mothers and health care providers toward pneumococcal conjugate vaccine in Bandung, West Java, Indonesia. Vaccine. 2013; 31(11): p. 1516–1522. 10.1016/j.vaccine.2013.01.007 23318150

[94] Andajani-Sutjahjo S, Manderson L. Stillbirth, neonatal death and reproductive rights in Indonesia. Reprod Health Matters. 2004; 12(24): p. 181–188. 15626208 10.1016/s0968-8080(04)24141-6

[95] Souganidis ES, Sun K, de Pee S, Kraemer K, Rah JH, Moench-Pfanner R, et al Relationship of maternal knowledge of anemia with maternal and child anemia and health-related behaviors targeted at anemia among families in Indonesia. Matern Child Health J. 2012; 16(9): p. 1913–1925. 10.1007/s10995-011-0938-y 22241619 PMC4101891

[96] Howard CT, de Pee S, Sari M, Bloem MW, Semba RD. Association of diarrhea with anemia among children under age five living in rural areas of Indonesia. J Trop Pediatr. 2007; 53(4): p. 238–244. 17463011 10.1093/tropej/fmm011

[97] Jayachandran S. Does Contraceptive Use Always Reduce Breast-feeding? Demography. 2014. 10.1007/s13524-014-0286-9 24659090

[98] Beegle K, Frankenberg E, Thomas D. Bargaining power within couples and use of prenatal and delivery care in Indonesia. Stud Fam Plann. 2001; 32(2): p. 130–146. 11449862 10.1111/j.1728-4465.2001.00130.x

[99] Nobles J, Frankenberg E. Mothers' community participation and child health. J Health Soc Behav. 2009; 50(1): p. 16–30. 19413132 10.1177/002214650905000102

[100] Sujarwoto S, Tampubolon G. Mother's social capital and child health in Indonesia. Soc Sci Med. 2013; 91: p. 1–9. 10.1016/j.socscimed.2013.04.032 23849232

[101] Februhartanty J, Usfar AA, Dianawati E, Fransisca DO, Roshita A, Fahmida U. Psychosocial care and nutritional status of children aged 6–36 months among patrilineal (Karo) and matrilineal (Minangkabau) households in Jakarta. Asia Pac J Clin Nutr. 2007; 16(2): p. 293–300. 17468086

[102] Van de Walle D. Sources of ethnic inequality in Viet Nam Journal of Development Economics. 2001; 65: p. 177–207.

[103] Li S, Zhu C, Feldman MW. Gender differences in child survival in contemporary rural China: a county study. J Biosoc Sci. 2004; 36(1): p. 83–109. 14989533 10.1017/s0021932004006121

[104] Krishnan A, Dwivedi P, Gupta V, Byass P, Pandav CS, Ng N. Socioeconomic development and girl child survival in rural North India: solution or problem? J Epidemiol Community Health. 2013; 67(5): p. 419–426. 10.1136/jech-2012-201846 23364028

[105] Jayaraj D, Subramanian S. Horizontal and vertical inequality: Some interconnections and indicators. Social Indicators Research. 2006; 75(1): p. 123–139.

[106] Marmot M, Friel S, Bell R, Houweling TA, Taylor S, Commission on Social Determinants of H. Closing the gap in a generation: health equity through action on the social determinants of health. Lancet. 2008; 372(9650): p. 1661–1669. 10.1016/S0140-6736(08)61690-6 18994664

[107] Cobham A, Summer A, Is inequality all about the tails? The Palma measure of income inequality, in CGD Working Paper 3432013, Center for Global Development: Washington D.C.

[108] UNICEF Indonesia. MDGs, equity and children: the way forward for Indonesia, 2012, UNICEF Indonesia: Jakarta.

[109] Starfield B. The hidden inequity in health care. Int J Equity Health. 2011; 10: p. 15 10.1186/1475-9276-10-15 21507245 PMC3094214

[110] UN Millennium Project. Who's got the power? Transforming health systems for women and children., Task Force for Child Health and Maternal Health, Editor 2005.

[111] United Nations. The Millennium Development Goals Report 2012, U. Nations, Editor 2012, United Nations: New York.

[112] Malqvist M, Sohel N, Do TT, Eriksson L, Persson LA. Distance decay in delivery care utilisation associated with neonatal mortality. A case referent study in northern Vietnam. BMC Public Health. 2010; 10: p. 762 10.1186/1471-2458-10-762 21144058 PMC3009650

[113] Okwaraji YB, Cousens S, Berhane Y, Mulholland K, Edmond K. Effect of geographical access to health facilities on child mortality in rural Ethiopia: a community based cross sectional study. PLoS One. 2012; 7(3): p. e33564 10.1371/journal.pone.0033564 22428070 PMC3299799

[114] Lohela TJ, Campbell OM, Gabrysch S. Distance to care, facility delivery and early neonatal mortality in Malawi and Zambia. PLoS One. 2012; 7(12): p. e52110 10.1371/journal.pone.0052110 23300599 PMC3531405

[115] Singh A, Padmadas SS, Mishra US, Pallikadavath S, Johnson FA, Matthews Z. Socio-economic inequalities in the use of postnatal care in India. PLoS One. 2012; 7(5): p. e37037 10.1371/journal.pone.0037037 22623976 PMC3356397

[116] Malqvist M, Hoa DT, Thomsen S. Causes and determinants of inequity in maternal and child health in Vietnam. BMC Public Health. 2012; 12(1): p. 641. 22883138 10.1186/1471-2458-12-641 PMC3534083

[117] Gabriele A, Schettino F. Child Malnutrition and Mortality in Developing Countries: Evidence from a Cross-Country Analysis. Analyses of Social Issues and Public Policy. 2008; 8(1): p. 53–81.

[118] Yuan B, Malqvist M, Trygg N, Qian X, Ng N, Thomsen S. What interventions are effective on reducing inequalities in maternal and child health in low- and middle-income settings? A systematic review. BMC Public Health. 2014; 14: p. 634 10.1186/1471-2458-14-634 24952656 PMC4083351

[119] Popkin BM, Adair LS, Ng SW. Global nutrition transition and the pandemic of obesity in developing countries. Nutr Rev. 2012; 70(1): p. 3–21. 10.1111/j.1753-4887.2011.00456.x 22221213 PMC3257829

[120] Vaezghasemi M, Ohman A, Eriksson M, Hakimi M, Weinehall L, Kusnanto H, et al The effect of gender and social capital on the dual burden of malnutrition: a multilevel study in indonesia. PLoS One. 2014; 9(8): p. e103849 10.1371/journal.pone.0103849 25153321 PMC4143167

[121] Dans A, Ng N, Varghese C, Tai ES, Firestone R, Bonita R. The rise of chronic non-communicable diseases in southeast Asia: time for action. Lancet. 2011; 377(9766): p. 680–689. 10.1016/S0140-6736(10)61506-1 21269677

[122] World Health Organization NCD country profile Indonesia, 2011, WHO: Geneva.

[123] DeWalt DA, A H. Health literacy and child health outcomes: a systematic review of the literature. Pediatrics. 2009 124 (3): p. S265–274. 10.1542/peds.2009-1162B 19861480

[124] Sanders LM, Federico S, Klass P, Abrams MA, B. D. Literacy and child health: a systematic review. Arch Pediatr Adolesc Med. 2009; 163(2): p. 131–140. 10.1001/archpediatrics.2008.539 19188645

[125] Singh A, Pallikadavath S, Ram F, Ogollah R. Inequalities in advice provided by public health workers to women during antenatal sessions in rural India. PLoS One. 2012; 7(9): p. e44931 10.1371/journal.pone.0044931 23028688 PMC3444494

[126] Guilmoto CZ. Son preference, sex selection, and kinship in Vietnam. Popul Dev Rev. 2012; 38(1): p. 31–54. 22833863 10.1111/j.1728-4457.2012.00471.x

[127] Zhou C, Wang XL, Zhou XD, T. H. Son preference and sex-selective abortion in China: informing policy options. Int J Public Health. 2012; 57(3): p. 459–465. 10.1007/s00038-011-0267-3 21681450

[128] Khera R, Jain S, Lodha R, Ramakrishnan S. Gender bias in child care and child health: global patterns. Arch Dis Child. 2014; 99(4): p. 369–374. 10.1136/archdischild-2013-303889 24344176

[129] Dalal K, Shabnam J, Andrews-Chavez J, Martensson LB, Timpka T. Economic empowerment of women and utilization of maternal delivery care in Bangladesh. Int J Prev Med. 2012; 3(9): p. 628–636. 23024852 PMC3445279

[130] Pokhrel S, R. S. Household decision-making on child health care in developing countries: the case of Nepal. Health Policy Plan. 2004; 19(4): p. 218–233. 15208278 10.1093/heapol/czh027

[131] World Economic Forum. The global gender gap report 2013, 2013, World Economic Forum: Geneva.

[132] WHO, Traditional birth attendants: a joint WHO/UNICEF/UNFPA statement, 1992, World Health Organization: Geneva.

[133] Malqvist M, Hoa DT, Liem NT, Thorson A, Thomsen S. Ethnic minority health in Vietnam: a review exposing horizontal inequity. Glob Health Action. 2013; 6: p. 1–19. 10.3402/gha.v6i0.19803 PMC3589437 23462107

[134] Yuan B, Qian X, Thomsen S. Disadvantaged populations in maternal health in China who and why? Glob Health Action. 2013; 6: p. 19542 10.3402/gha.v6i0.19542 23561030 PMC3617641

[135] Sibley LM, Sipe TA, Brown CM, Diallo MM, McNatt K, N. H. Traditional birth attendant training for improving health behaviours and pregnancy outcomes. Cochrane Database of Systematic Reviews. 2007; 3(CD005460). 10.1002/14651858.CD005460.pub2 17636799

[136] Fatmi Z, Gulzar AZ, A K. Maternal and newborn care: practices and beliefs of traditional birth attendants in Sindh, Pakistan. East Mediterr Health J. 2006; 11(1–2): p. 226–234. 16532692

[137] Syed U, Khadka N, Khan A, S W. Care-seeking practices in South Asia: using formative research to design program interventions to save newborn lives. J Perinatol. 2008; 28(Suppl2). 10.1038/jp.2008.165 19057572

[138] Brockerhoff M, Hewett P. Inequality of child mortality among ethnic groups in sub-Saharan Africa. Bull World Health Organ. 2000; 78(1): p. 30–41. 10686731 PMC2560588

[139] Burgard SA, Treiman DJ. Trends and racial differences in infant mortality in South Africa. Soc Sci Med. 2006; 62(5): p. 1126–1137. 16135394 10.1016/j.socscimed.2005.07.025

[140] Moher D, Fortin P, Jadad AR, Juni P, Klassen T, Le Lorier J, et al Completeness of reporting of trials published in languages other than English: implications for conduct and reporting of systematic reviews. Lancet. 1996; 347(8998): p. 363–366. 8598702 10.1016/s0140-6736(96)90538-3

[141] Juni P, Holenstein F, Sterne J, Bartlett C, Egger M. Direction and impact of language bias in meta-analyses of controlled trials: empirical study. Int J Epidemiol. 2002; 31(1): p. 115–123. 11914306 10.1093/ije/31.1.115

[142] Bryce J, Arnold F, Blanc A, Hancioglu A, Newby H, Requejo J, et al Measuring coverage in MNCH: new findings, new strategies, and recommendations for action. PLoS Med. 2013; 10(5): p. e1001423 10.1371/journal.pmed.1001423 23667340 PMC3646206

[143] Ruhago GM, Ngalesoni FN, Norheim OF. Addressing inequity to achieve the maternal and child health millennium development goals: looking beyond averages. BMC Public Health. 2012; 12: p. 1119 10.1186/1471-2458-12-1119 23270489 PMC3543393

[144] Braveman P, Gruskin S. Defining equity in health. J Epidemiol Community Health. 2003; 57(4): p. 254–258. 12646539 10.1136/jech.57.4.254 PMC1732430

[145] Waage J, Banerji R, Campbell O, Chirwa E, Collender G, Dieltiens V, et al The Millennium Development Goals: a cross-sectoral analysis and principles for goal setting after 2015 Lancet and London International Development Centre Commission. Lancet. 2010; 376(9745): p. 991–1023. 10.1016/S0140-6736(10)61196-8 20833426 PMC7159303

