# Supplementary material for: Millennium Development Goal Four and Child Health Inequities in Indonesia: A Systematic Review of the Literature
Source: PLoS One. 2015 May 5;10(5):e0123629. doi: 10.1371/journal.pone.0123629 (PMC4420469; doi:10.1371/journal.pone.0123629)
Supplement: S1 Search Syntax — (PDF) [file pone.0123629.s002.pdf]

Appendix 1: Detailed Search Syntax for Pubmed Database.

((((((((((((((((((((((under-five) OR under-5) OR child) OR infant) OR neonate) OR newborn) OR pediatric) OR pediatrics)) AND ((((((((((((((((((((((((child health) OR infant health) OR neonatal health) OR newborn health) OR health status) OR child survival) OR under-five mortality) OR under-five mortality) OR child mortality) OR infant mortality) OR neonatal mortality) OR perinatal mortality) OR postnatal mortality) OR newborn mortality) OR under-five death) OR under-5 death) OR child death) OR infant death) OR neonatal death) OR perinatal death) OR postnatal death) OR newborn death) OR stillbirth) OR stillborn)) AND (((governance) OR government) OR administration) OR policy) OR policy making) OR public policy) OR reform) OR political) OR right) OR rights) OR economic) OR economics) OR macroeconomic) OR economic policy) OR economic crisis) OR GDP) OR gross domestic product) OR sociopolitical) OR socio political) OR sociocultural) OR socio cultural) OR social) OR social policy) OR social security) OR social insecurity) OR social care) OR social protection) OR social housing) OR national) OR regional) OR community) OR neighborhood) OR household) OR family) OR families) OR labor market) OR price) OR inflation) OR welfare) OR services) OR land use) OR public health) OR family planning) OR child protection) OR pension) OR culture) OR value) OR value system) OR integration) OR beliefs) OR social network) OR social capital) OR custom) OR tradition) OR language) OR empowerment) OR decentralization)) OR (((((((((((((((((immunization) OR immunity) OR vaccination) OR vaccine) OR measles) OR DTP) OR diphtheria) OR tetanus) OR pertussis) OR HIB vaccine) OR haemophillius influenza type b) OR polio) OR poliomyelitis) OR BCG vaccine) OR Bacillus Calmette-Guerin) OR tuberculosis) OR TB)) OR ((((((((((((((((((((((((((((((((((((((maternal) OR maternal age) OR birth interval) OR pregnancy interval) OR birth order) OR birth rank) OR first birth) OR parity) OR birth attendant) OR midwife) OR trained attendant) OR skilled attendant) OR traditional birth attendant) OR traditional medicine) OR healer) OR child birth) OR childbearing) OR pregnancy outcome) OR type of delivery) OR place of delivery) OR home delivery) OR birth choice) OR delivery complications) OR child development) OR pregnancy complications) OR pregnancy history) OR high risk pregnancy) OR adolescent pregnancy) OR KMC) OR kangaroo mother care) OR PMTCT) OR preventing mother to child transmission of HIV) OR drug abuse) OR IMCI) OR integrated management of childhood illnesses) OR postnatal care services) OR parental investment) OR parental competence)) OR ((((((((((((((((((((((((((nutrition) OR diet) OR nutrition disorder) OR breast feeding) OR breastfeeding) OR early initiation of breastfeeding) OR exclusive breastfeeding) OR introduction solid semi-solid soft foods) OR supplementary feeding) OR vitamin a supplementation) OR micronutrients) OR iron) OR folic acid) OR ORT) OR oral rehydration therapy) OR continued feeding) OR iodized salt) OR food safety) OR food contamination) OR food insecurity) OR low birth weight) OR stunting) OR wasting) OR underweight) OR malnutrition) OR caloric intake)) OR ((((((((((((((environment) OR water) OR water quality) OR drinking water) OR fluoridation) OR purification) OR sanitation) OR disposal) OR toilet) OR hygiene) OR pollution) OR contamination) OR smoking) OR bed net)) OR ((disease) OR cause) OR childhood disease) OR childhood disorder) OR cause of death) OR health damage) OR exposure) OR determinant) OR determinants) OR diarrhea) OR diarrheal) OR tetanus) OR pneumonia) OR preterm) OR premature) OR prematurity) OR birth complications) OR asphyxia) OR sepsis) OR congenital abnormalities) OR malformation) OR birth defect) OR pertussis) OR measles) OR meningitis) OR malaria) OR infection) OR infectious disease) OR non communicable disease) OR injury) OR injuries) OR accident) OR HIV AIDS) OR human immunodeficiency virus) OR acquired immunodeficiency syndrome) OR ARI) OR acute respiratory infection) OR neonatorum) OR anemia) OR sudden infant death syndrome) OR SIDS) OR fever)) OR ((socioeconomic) OR socioeconomic status) OR social class) OR upper class) OR middle class) OR lower class) OR low resource setting) OR demographic) OR demography) OR occupation) OR vocation) OR profession) OR job) OR job sector) OR unemployed) OR employed) OR employment status) OR worker) OR workplace) OR farmer) OR informal) OR poverty) OR poor) OR indigent) OR income) OR high income) OR middle income) OR low income) OR standard of living) OR expenditure) OR residence) OR rural) OR urban) OR urbanization) OR slum) OR remote) OR homeless) OR mobility) OR residential mobility) OR migration) OR migration) OR migrant) OR immigrant) OR immigration) OR emigrant) OR emigration) OR western area) OR child labor) OR child abuse) OR orphan) OR illegitimacy) OR illegitimate) OR disability) OR handicapped) OR married) OR unmarried) OR divorce) OR one parent)) OR (((((education) OR schooling) OR literacy) OR illiteracy) OR educational status)) OR ((((((((((((((((((((((gender) OR identity) OR sex role) OR ethnicity) OR ethnic) OR ethnic group) OR minority) OR indigenous) OR tribal) OR native) OR religion) OR Islam) OR Muslim) OR Hinduism) OR Hindu) OR caste) OR Christianity) OR Christian) OR discrimination) OR marginal) OR marginalization) OR stigma)) OR ((health care) OR health system) OR health services) OR health center) OR delivery of health care) OR health care disparities) OR health care access) OR geographical constraints) OR infrastructure) OR transportation) OR distance) OR health personnel attitude) OR health worker attitude) OR cultural competence) OR social norms) OR health resources) OR human resource) OR supplies) OR equipment) OR devices) OR manpower) OR facilities) OR insurance) OR insured) OR uninsured) OR insurance status) OR insurance coverage) OR universal coverage) OR out of pocket) OR financial constraints) OR costs) OR expensed) OR expenditure) OR child care) OR child health services) OR prevention) OR preventive) OR health policy) OR Millennium Development Goal Four) OR Millennium Development Goal 4) OR MDG Four) OR MDG 4)) OR ((((((((((((((equity) OR inequity) OR inequities) OR equality) OR inequality) OR inequalities) OR disparity) OR disparities) OR disadvantaged group) OR disadvantaged population) OR vulnerable group) OR vulnerable population) OR sensitive group) OR sensitive population)) AND Indonesia Filters: **Publication date from 1995/01/01 to 2014/05/15; Humans; English**
